# Competition between O–H and S–H Intermolecular Interactions in Conformationally Complex Systems: The 2-Phenylethanethiol and 2-Phenylethanol Dimers

**DOI:** 10.1021/acs.jpclett.4c00903

**Published:** 2024-05-20

**Authors:** Fernando Torres-Hernández, Paul Pinillos, Wenqin Li, Rizalina Tama Saragi, Ander Camiruaga, Marcos Juanes, Imanol Usabiaga, Alberto Lesarri, José A. Fernández

**Affiliations:** † Departamento de Química Física, Facultad de Ciencia y Tecnología, Universidad del País Vasco (UPV/EHU), Barrio Sarriena s/n, Leioa 48940, Spain; § Departamento de Química Física y Química Inorgánica, Facultad de Ciencias - I.U. CINQUIMA, Universidad de Valladolid, Paseo de Belén, 7, E-47011 Valladolid, Spain

## Abstract

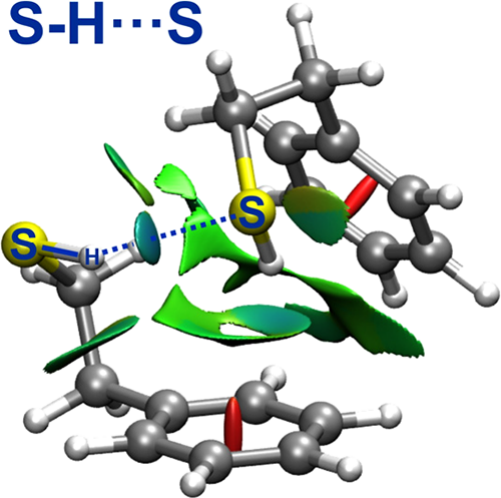

Noncovalent interactions involving sulfur centers play a relevant role in biological and chemical environments. Yet, detailed molecular descriptions are scarce and limited to very simple model systems. Here we explore the formation of the elusive S–H···S hydrogen bond and the competition between S–H···O and O–H···S interactions in pure and mixed dimers of the conformationally flexible molecules 2-phenylethanethiol (PET) and 2-phenylethanol (PEAL), using the isolated and size-controlled environment of a jet expansion. The structure of both PET–PET and PET–PEAL dimers was unraveled through a comprehensive methodology that combined rotationally resolved microwave spectroscopy, mass-resolved isomer-specific infrared laser spectroscopy, and quantum chemical calculations. This synergic experimental–computational approach offered unique insights into the potential energy surface, conformational equilibria, molecular structure, and intermolecular interactions of the dimers. The results show a preferential order for establishing hydrogen bonds following the sequence S–H···S < S–H···O ≲ O–H···S < O–H···O, despite the hydrogen bond only accounting for a fraction of the total interaction energy.

The structure of biological macromolecules is the result of a subtle balance between many different types of intra- and intermolecular interactions. Among them, the hydrogen bond plays the most determinant role not only due to its strength but also because it is highly directional, forcing the functional groups to adopt positions maximizing their interaction. Therefore, the extensive literature existing around such interactions is not surprising, especially in condensed phases.^1−3^ Alternatively, jet-cooled spectroscopies permit analyzing specific intermolecular interactions in the gas phase, while simultaneously removing matrix or solvent effects.^4^ This strategy has enabled the characterization of numerous chemical systems, yielding valuable information regarding the intrinsic nature of the hydrogen bond and other weak molecular forces. Still, most such studies dealt with the most paradigmatic OH···O hydrogen bond,^3^ leaving aside other important interactions, such as those involving chalcogen centers,^5^ in particular sulfur-centered hydrogen bonds.^6^

The molecular information obtained from competing interactions is of utmost importance to understand the nature of the hydrogen bond and to benchmark quantum-chemical computations.^7^ Intuitively, the more electronegative the acceptor/donor atoms, the stronger the hydrogen bond. However, additional parameters such as atomic size and polarizability are also relevant.^8^ Recent studies have demonstrated that under some circumstances sulfur is able to form hydrogen bonds as strong as oxygen or even stronger.^9−12^ In addition, it is not clear what type of hydrogen bond would be preferred in a mixture of species containing thiol (R–SH) and alcohol (R–OH) groups. Previous studies in the gas phase using H_2_S–methanol adducts demonstrated that the S–H···O hydrogen bond is preferred by a narrow margin,^13^ but the energy difference is small enough to lie in the gray zone of the accuracy of the computational methods (typically a few kJ mol^–1^). A similar hydrogen bond was observed for 2-phenylethanethiol-diethyl ether, but not in 2-phenylethanethiol-water.^14^ Other molecular studies have mostly observed thiols as proton acceptors, especially in O–H···S,^15−18^ N–H···S,^9,11,19^ and C–H···S^20,21^ hydrogen bonds. Thiol dimerization studies will thus contribute to the description of thiols as proton donors in S–H···S^22−25^ and other weak sulfur interactions (S–H···N,^26^ S–H···π,^27−29^ etc.), which are far less investigated.

Most of the gas-phase cluster studies have been carried out on small rigid systems, trying to isolate simple individual interactions. This approach permits a more precise characterization but is difficult to extrapolate to biological systems, in which several interactions compete to give the final shape to the aggregates or biopolymers. Motivated by the lack of information on competing interactions of O–H/S–H···O/S in conformationally complex systems, we explore here the homodimer of 2-phenylethylthiol (PET–PET) and the heterodimer between PET and 2-phenylethanol (PET–PEAL). Both monomers present an aromatic ring and a polar group connected through a flexible ethyl group and multiple binding sites at the ring and the polar groups (Figure S1). Moreover, the low interconversion barriers in the three internal rotors (at C_α_, C_β_, and S/O) may also result in transient chirality, producing (+/−) axial enantiomers connected by quantum tunnelling (Figure S2).^25,30,31^ Previous studies on PEAL aggregation demonstrated unexpected conformational complexity,^32^ as three different isomers were observed in the gas phase for the PEAL–PEAL dimer (Figure S3). Characterization of the PET–PET and PET–PEAL dimers will allow us to determine how the small energy difference between S–H···S and O–H···O interactions affects the formation of aggregates and how the changes in the potential energy surface influence conformational multiplicity and the balance of noncovalent interactions. Unlike in previous studies, formation of the hydrogen bond accounts only for a fraction of the total interaction energy, making it difficult to anticipate the dimer structure. A description of the preferred hydrogen bond interactions in the dimer structure may in turn help us to understand the structural properties of larger clusters.

The gas-phase acidity of aliphatic alcohols is typically lower than for their thiol counterparts;^33^ so, in principle, heterodimers based predominantly on S–H···O hydrogen bonds should be formed. However, we show that the formation of the dimer will balance not only the thiol/alcohol group but also the rest of the molecular interactions in the dimer.

We address all of these questions through comprehensive high-resolution experiments, combining rotational and vibronic information obtained from microwave (MW) and mass-resolved excitation spectroscopy (MRES) techniques. While MW has an unparalleled structure-resolving power,^34^ MRES yields mass- and isomer-selected information on S–H/O–H stretchings that can be directly correlated with the strength of the hydrogen bonds,^4,7^ offering full insight into the electronic structure and noncovalent interactions in the dimers.

The jet-cooled electronic REMPI spectra of PET, PEAL, PET–PET, and PET–PEAL in Figure S4 provided compelling evidence of conformational multiplicity. We confirmed the presence of two isomers in the gas phase for the monomers, as previously reported (Figure S5).^35−39^ The global minimum (Ggπ) has a skew (*gauche*–gauche) side chain characterized by an O–H···π or S–H···π intramolecular hydrogen bond. The second isomer is very close in stability (ca. 4.8 and 2.9 kJ/mol, respectively,^35−39^ above the global minimum) but differs in the antiperiplanar (At in PEAL) or *gauche* (Ag in PET) orientation of the terminal alcohol or thiol groups. The ion-dip infrared spectra (IDIRS) were recorded by probing the 0_0_^0^ origin bands. Well-resolved vibrational spectra were obtained for PEAL (O–H stretches appeared at 3624 and 3678 cm^–1^ for Ggπ and At, respectively), but the S–H stretching mode was too weak for detection (Figure S6).

Molecular dimerization into PET–PET and PET–PEAL results in a flat and dimensionally complex potential energy surface. Assuming formation of a S–H···S hydrogen bond for the PET–PET dimer, the four Ggπ(+/−) and Ag(+/−) monomers and the two proton acceptor (Lp+/−) lone pairs may generate 4^2^ × 2 stereoisomer classes or 16 families of enantiomers, each multiplied by a number of plausible secondary interactions between the thiol groups and the two rings. Similar arguments hold for the PET–PEAL dimer, but with the added complexity of the combination of two different molecules. The conformational space of the PET dimers thus required extensive computational exploration using dispersion-corrected density functional theory (DFT). The computational procedures are described in the Supporting Information (SI) and included hybrid (B3LYP^40^) and double-hybrid (B2PLYP^41^) DFT methods and D3^42^ empirical dispersion corrections (Becke-Johnson damping function)^43^ combined with a triple-ζ basis set. The most stable structures for the PET–PET and PET–PEAL dimers are collected in Tables S1–S4 and Figures S7 and S8.

Dimerization caused a red-shift in the origin band in the MRES spectrum of both PET–PET and PET–PEAL (Figure S4). Assuming that the 0_0_^0^ transition is the red-most band, it appears at 37,397 cm^–1^ for PET–PET and at 37,374 cm^–1^ for PET–PEAL. IDIR vibrational spectra were then recorded probing all the bands in the REMPI spectrum of PET–PET, always obtaining the spectrum in Figure 1. Comparison with the computational predictions (Figures 1A and S9) confirmed the assignment of the single experimental PET–PET isomer to the global minimum. Formation of an S–H···S–H···π hydrogen bond network results in an increase in the intensity of the stretching bands of both S–H groups, making their observation possible at 2540 and 2567 cm^–1^, although still with a low intensity. Part of this low intensity must be due to the decrease in power of the OPO used as IR source. One must take into account that no normalization procedure was used to correct for the changes in output power of this laser.

**Figure 1:**
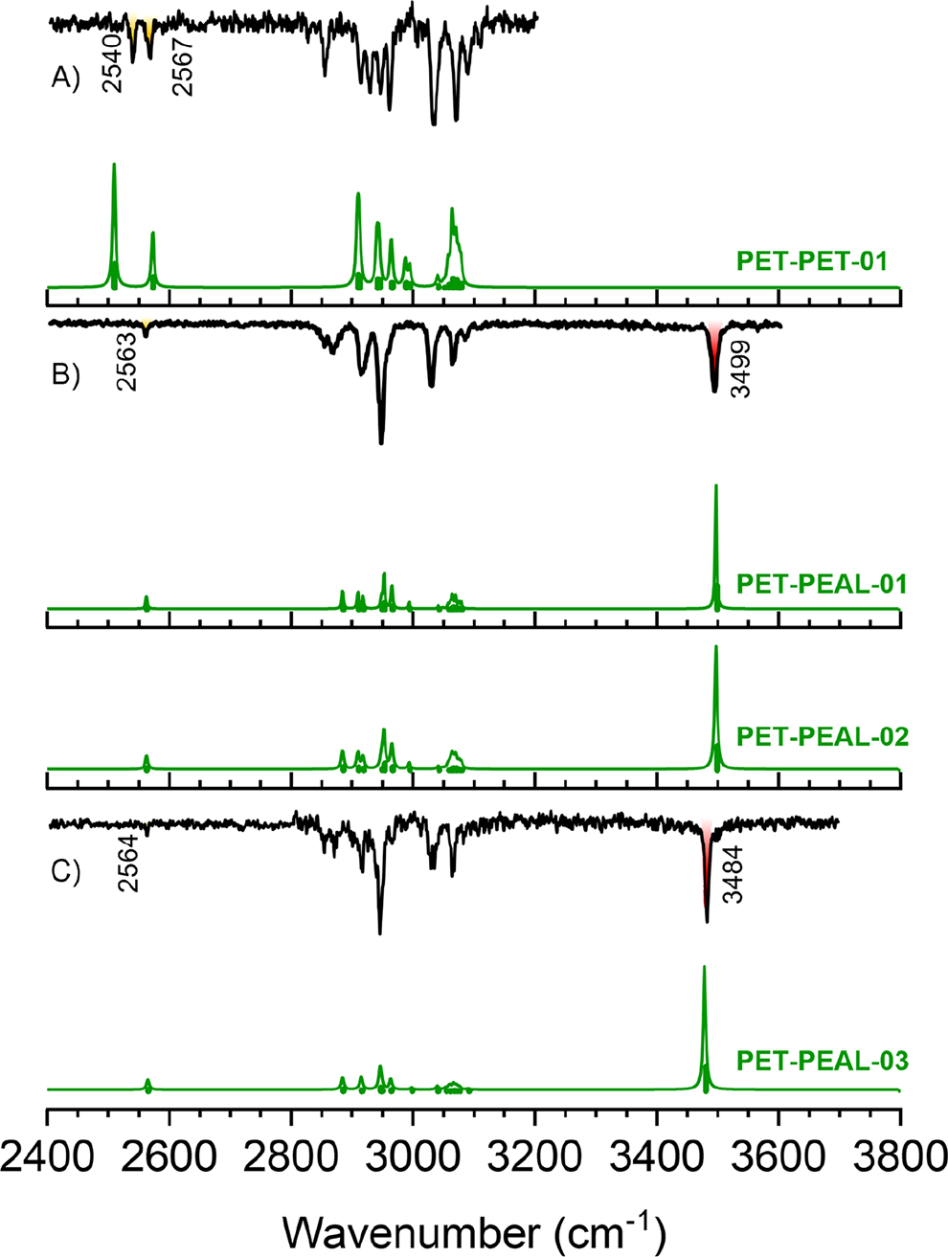
Comparison between IDIR and simulated spectra for the isomers found in the jet expansion of (A) PET–PET (UV laser tuned at 37418 cm^–1^); (B) PET–PEAL experimental isomer 1 (UV laser tuned at 37522 cm^–1^) and (C) PET–PEAL experimental isomer 2 (UV laser tuned at 37448 cm^–1^). From the vibrational point of view, PET–PEAL experimental isomer 1 could be assigned either to the global minimum or to the second most stable structure, as their predicted IR spectra are nearly identical. A correction factor of 0.963 was applied to account for the anharmonicity.

The calculations predicted with relative accuracy the position of the bands, but they suggested a spacing larger than that observed in the experimental spectrum. Regarding the CH stretches, the calculations correctly predicted their position, although, as is typical in this kind of system, they were not able to correctly reproduce the full experimental spectrum.

The detection of the PET–PET dimer was independently confirmed by using microwave spectroscopy. Once the transitions for the two PET monomers were removed from the rotational spectrum, weaker signals originated by a new asymmetric rotor were positively identified (Figure 2). The spectral analysis used a semirigid rotational Hamiltonian^44^ and provided accurate rotational and centrifugal distortion parameters, shown in Table 1. Further comparison with the predicted spectroscopic parameters unequivocally established the detection of the PET–PET global minimum, denoted as Ggπ+Ggπ–Lp– in Tables 1, S1, and S2.

**Figure 2:**
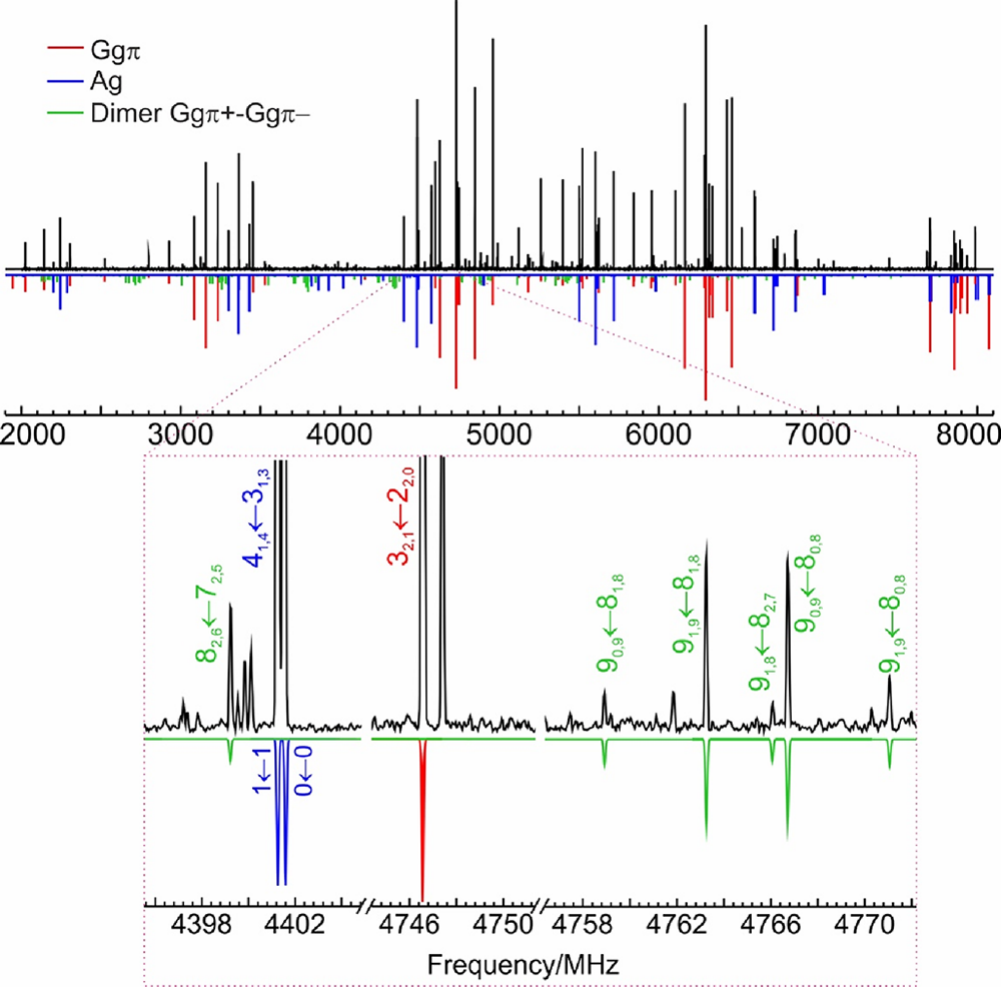
Microwave spectrum of PET (positive black trace) and simulations based on the fitted parameters of the two isomers of the monomer^39^ (Ggπ and Ag, red and blue traces) and the PET–PET dimer Ggπ+Ggπ–Lp– in Table 1 (green trace).

**Table 1:**
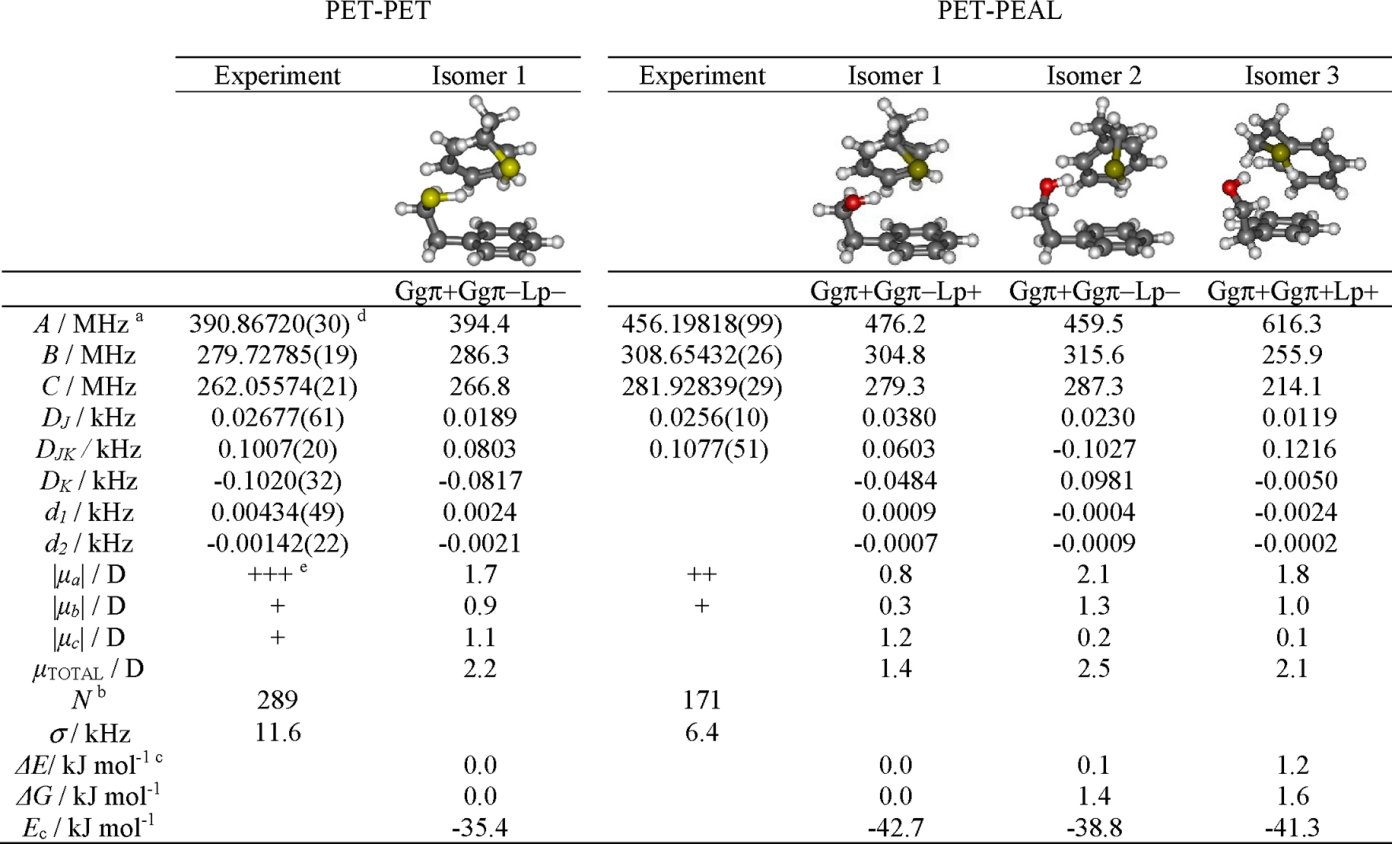
Rotational Parameters for the PET–PET and PET–PEAL Dimers Compared to the Theoretical Predictions at the B2PLYP-D3(BJ)/def2-TZVP Level

a Rotational constants (*A*, *B*, *C*), Watson’s S-reduction (I^r^ representation) centrifugal distortion constants (*D*_*J*_, *D*_*JK*_, *D*_*K*_, *d*_1_, *d*_2_) and electric dipole moments (μ_α_, α = *a*, *b*, *c*).

b Number of transitions (*N*) and rms deviation (σ) of the rotational fit.

c Relative electronic energies (Δ*E*) with zero-point correction, Gibbs energy (Δ*G*, 298 K, 1 atm), and complexation energies (*E*_c_).

d Standard errors in parentheses in units of the last digit.

e One or more plus signs (+) qualitatively indicate the relative intensity of the observed rotational transitions.

In the case of the PET–PEAL heterodimer, the IR-UV experiment demonstrated the existence of two different isomers (Figure S10). The IDIR spectrum in Figures 1 and S11 is very similar for both isomers, with red-shifted O–H stretches, a comparable C–H fingerprint, and very weak S–H stretching bands. The first experimental spectrum could be assigned either to the predicted global minimum isomer 1 (Ggπ+Ggπ–Lp+) or to the second most stable isomer 2 (Ggπ+Ggπ–Lp−), characterized by O–H···S–H···π sequential hydrogen bonds. Both isomers present very similar computed IR spectra and, in addition, are isoenergetic. Therefore, it is not possible to reach a univocal assignment. The second experimental spectrum was assigned to the calculated isomer 3 (Ggπ+Ggπ+Lp+) slightly above the global minimum (ca. 1.2 kJ mol^–1^; Tables 1, S3, and S4). This structure presents a similar hydrogen bond network but with the proton-acceptor molecule adopting a different conformation. The impact of the structural changes in the spectrum is small and results in a modest shift in the O–H/S–H stretches. Still, such a small shift is captured nicely in the simulation of isomer 3. From a computational point of view, many other structures containing such hydrogen bond networks exist within a narrow energy window, but they offer an inferior vibrational match (Figure S11).

A further survey of the microwave spectrum of PET–PEAL in Figure S12 completed the structural assignment. The rotational data detected a single isomer, which could be reproduced with a semirigid rotor model. The rotational parameters and electric dipole moments in Table 1 confirmed the detection of isomer 2 (Ggπ+Ggπ–Lp−), which can then be identified as the global minimum of PET–PEAL. The absence of isomer 1 is probably associated with a collisional low-energy interconversion pathway to the real global minimum.^45^ Rotational detection of isomer 3 dimer failed, which could be attributed to their lower dipole moments or different expansion conditions in the rotational jet (neon) vs the laser experiments (helium).

Figure 3 summarizes the structures of the adducts assigned in this work, together with those of the PEAL–PEAL dimer prototype^32^ based on the O–H···O hydrogen bond. The PET–PET dimer permitted the characterization of the less frequent S–H···S hydrogen bond,^22−25^ while in PET–PEAL both O–H···S^15−18^ and S–H···O^13,14,46^ hydrogen bonds were plausible. However, both experiment and theory confirmed the energetic preferences for the O–H···S interaction by ca. 4 kJ mol^–1^ (Tables S3 and S4). This result is consistent with most of the gas-phase observations, but the small energy differences may favor a conformational equilibrium shift to the O–H···S hydrogen bond for specific electronic environments.^13,14,46^ For this reason, generalization of these observations will require additional experiments. In the three PET–PET, PET–PEAL, and PEAL–PEAL aromatic dimers, the aggregates adopt a nonstacked cooperative hydrogen bonded structure, which is the result of the competition between the primary hydrogen bond and weaker secondary S/O–H···π and C–H···π interactions. This situation is reminiscent of the conformational equilibrium in the benzyl mercaptan^31^ and benzyl alcohol^25^ dimers. Replacement of the O atom by a S atom does not alter substantially the preferred conformation of the aggregates but results in a considerable increase of the (X···H, X = O, S) hydrogen bond distance, from 1.88 Å in PEAL–PEAL global minimum, to 2.45 Å in PET–PEAL, and to 2.88 Å in PET–PET (structural data in Tables S1–S4). The existence of 3p orbitals in the S atom conditions the kind of interactions that the sulfur atom can produce. The larger size of this orbital compared with the 2s/2p of the oxygen atom has several effects. First, a larger size means larger polarizability, and therefore, S is more prone to produce stronger interactions with other polarizable groups. Second, the larger size also means longer interaction distances in hydrogen bonds. The repulsion between the interacting molecules grows faster than in the case of oxygen, but the overlap between orbitals is also larger at longer distances.^13^ Nevertheless, as the electron density in the 3p orbital is smaller, the result is a smaller charge transfer component than in the case of O–H···O interaction.^13^ Altogether, the balance of the interactions generally produces stronger O–H···O than S–H···S hydrogen bonds.

**Figure 3:**
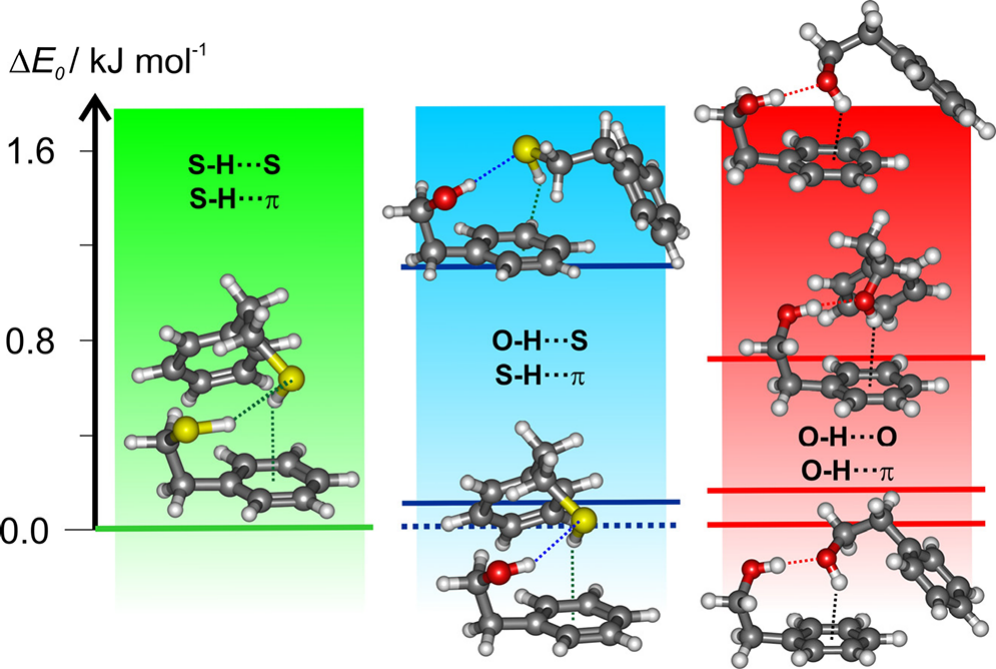
Experimentally observed dimers of PET–PET (left), PET–PEAL (center), and PEAL–PEAL^31^ (right) and the lowest predicted electronic energies for the three adducts (B2LYP-D3(BJ)/def2-TZVP, Tables S1–S4).

The presence of a variety of intermolecular interactions forming the dimers is revealed in the noncovalent interaction analysis^47^ of Figure 4, which shows the primary hydrogen bond accompanied by a secondary OH···π/SH···π interaction and other dispersion-dominated weaker C–H···π contacts. Comparison between PET–PET and PEAL–PEAL shows changes in the delicate balance between interactions, favoring the adoption of a different set of stereoisomers.

**Figure 4:**
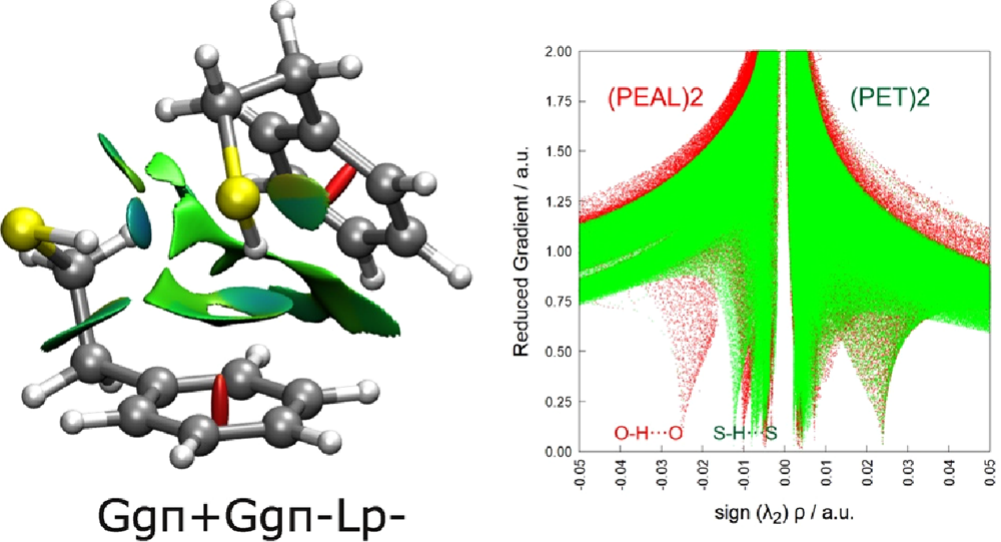
Noncovalent interaction analysis^47^ for PET–PET (left panel), mapping the regions of weak attractive interactions between the two molecules (green-blue colors), and representation of the reduced electronic density gradient (right panel) for PET–PET (green) and PEAL–PEAL (red), showing the weaker character of the S–H···S interaction compared to the O–H···O hydrogen bond.

Perhaps the most interesting observation is related to the mixed PET–PEAL dimer. Aliphatic alcohols have higher proton affinities than the equivalent thiols. Thus, one might expect the S–H···O interaction to be predominant in the formation of the heterodimer. However, only the O–H···S interaction was observed. Likewise, all theoretical predictions point to a higher stability of the O–H···S species. The most stable S–H···O species appears at 4.3 kJ/mol (B3LYP-D3(BJ)) above the global minimum and presents a conformation similar to that of the PET–PET dimer (see Figures 3 and S8). We may therefore conclude that the influence of the acceptor heteroatom in the structure of the dimer is larger than that in the donor. Perhaps this is because the acceptor presents additional interactions with the aromatic ring and other parts of both molecules. The strength of the S–H···S vs O–H···O interactions can also be gauged through the representation in Figure 4 of the reduced electronic density gradient  vs the signed electronic density (sign(λ_2_)ρ, with λ_2_ the second eigenvalue of the electronic density Hessian). The comparison of the PET–PET and PEAL–PEAL reduced gradients shows a critical point for PEAL–PEAL at more negative abscissas, confirming the expected stronger character of the O–H···O vs the S–H···S interaction.

Further investigation into the nature of the physical forces controlling the noncovalent interactions was obtained through energy decomposition analysis, using symmetry-adapted perturbation theory (SAPT). The results in Table 2 reflect not only the decrease of binding energies in PET–PET compared to PET–PEAL and PEAL–PEAL but also a redistribution of electrostatic and dispersion components associated with the presence of oxygen or sulfur heteroatoms. The alcohol homodimer is the only adduct with prevailing electrostatic forces, while both PET–PET and PET–PEAL are dominated by dispersion forces.

**Table 2:** Results from Symmetry Adapted Perturbation Theory (SAPT2 + 3(CCD)/aug-cc-pVDZ) Binding Energy Decomposition for PET–PET, PET–PEAL, and PEAL–PEAL, Comparing the Magnitude of the Electrostatic and Dispersion Contributions^a^

			PEAL–PEAL		
	PET–PET	PET–PEAL	I	II	III
Δ*E*_Electrostatic_	–39.5(33.4%)^b^	–45.0(37.8%)	–63.4(43.6)	–68.4(44.4%)	–63.5(44.0%)
Δ*E*_Dispersion_	–65.0(54.9%)	–57.7(48.4%)	–59.3(40.8%)	–59.8(38.8%)	–58.1(40.3%)
Δ*E*_Induction_	–13.9(11.7%)	–16.4(13.8%)	–22.7(15.6%)	–25.8(16.7%)	–22.7(15.7%)
Δ*E*_Exchange_	84.2	79.3	98.8	105.1	96.7
Δ*E*_Total_	–34.2	–39.8	–46.6	–48.9	–47.6

a All values are in kJ mol^–1^.

b Energetic contributions and relative percentage in parentheses with respect to the total attractive energy.

In conclusion, the combination of computational, vibronic, and rotational data constitutes a powerful methodology for the investigation of noncovalent interactions on weakly bound systems of increasing complexity. The case of the PET and PEAL dimers are representative of larger flexible biological systems, including aromatic and polar chalcogen groups, simultaneously offering adjustable conformational balances involving different noncovalent interactions. The replacement of oxygen by sulfur in one of the monomers impacts the strength and directionality of the primary hydrogen bond interaction, but its reduction in strength seems to be compensated by chalcogen-ring and interring secondary interactions, so the net force balance in this case produces similarly ring-tilted structures involving the chalcogen and ring forces. At the same time, while the dispersion component notably increases with the number of sulfur atoms, the hydrogen bond control is maintained in the dimerization of the PET/PEAL monoaryl compounds, all departing from the π-stacked geometries observed in biaryl compounds.^48,49^ Future work will assess the influence of noncovalent interactions in the conformational equilibria of heavier chalcogens and larger biaryl clusters.

## Supporting Information Available

Experimental and computational methods; Figures S1–S15 and Tables S1–S9 with computational results, list of the observed rotational transitions, and theoretical Cartesian coordinates (PDF)
