# Supplementary material for: Competition between O–H and S–H Intermolecular Interactions in Conformationally Complex Systems: The 2-Phenylethanethiol and 2-Phenylethanol Dimers
Source: J Phys Chem Lett. 2024 May 20;15(21):5674–80. doi: 10.1021/acs.jpclett.4c00903 (PMC11145646; doi:10.1021/acs.jpclett.4c00903)
Supplement: Supplementary file 1 — jz4c00903_si_001.pdf [file jz4c00903_si_001.pdf]

Competition Between O-H and S-H Intermolecular Interactions in Conformationally Complex Systems: The 2-Phenylethanethiol and 2-Phenylethanol Dimers

SUPPORTING INFORMATION

Fernando Torres-Hernández,[†] *Paul Pinillos,*[†] *Wenqin Li,*[§] *Rizalina Tama Saragi,*[§‡] *Ander Camiruaga,*[†] *Marcos Juanes,*[§] *Imanol Usabiaga,*[†] *Alberto Lesarri,*[§]* *José Andrés Fernández,*[†]*

[†]Departamento de Química Física, Facultad de Ciencia y Tecnología, Universidad del País Vasco, Apartado 644, E-48080 Bilbao (Spain)
[§]Departamento de Química Física y Química Inorgánica, Facultad de Ciencias - I.U. CINQUIMA, Universidad de Valladolid, Paseo de Belén, 7, E-47011 Valladolid (Spain)
[‡]Present address: Argonne National Laboratory, 9700 S. Cass Avenue, Lemont, IL 60439

Experimental and computational methods

Mass-Resolved Excitation Spectroscopy. The experimental set up has been described in detail in previous publications,[1] and consists of an in-house designed linear time-of-flight (ToF) mass spectrometer; two systems of Nd/YAG laser + dye laser + doubling unit (Quantel Brilliant B + Fine adjustment and Quantel Qsmart 850 + Qscan) and an OPO system (LaserVision) pumped by a Nd/YAG laser (Continuum mod Surelite), together with the required electronics for control and signal acquisition.

Briefly, the sample (2-phenylethanethiol and/or 2-phenylethanol) was deposited in a sample holder and inserted in the gas line feeding a pulsed valve (Jordan Inc.) attached to the ionization chamber of an in-house designed mass spectrometer. At each aperture of the valve, operated at 10 Hz, a supersonic expansion was created, producing an adiabatic cooling of the molecules down to 2-5 K of rotational temperature and 50 – 100 K of vibrational temperature. Under such conditions, the molecules aggregate. Either He or Ne were used as buffer gas at typical pressures between ~2-5 bar. The species populating the beam were excited with UV photons that first excited the $S_1 \leftarrow S_0$ electronic transition and then ionize the molecules. The electric field, created by a pair of extraction/repulsion plates with 400 V of voltage difference between them, sent the ions towards the detector, accelerating them according to their charge-to-mass ratio. Before reaching the field-free flight path, the ions were further accelerated by an acceleration plate. During the travel through the field-free region of the mass spectrometer, the ions segregate according to their mass, arriving to the detector at different times.

The detector consisted of a pair of MCP plates that produced a ~4 mV pulse per ion. The signal produced by the ions was collected with the aid of a digital oscilloscope (Tektronix TDS 3032), integrated and routed to the same computer that controls the whole experiment. Synchronization between the lasers, valve and oscilloscope was handled using three SRS 645 (Standford Research Systems) pulse generators.

In the 2-color resonance enhanced multiphoton ionization (REMPI) experiments, the excitation laser was scanned through the region of interest, while the total ion current in a given mass-channel was recorded. In this way, the mass-resolved electronic excitation spectrum of the molecules of interest was recorded, with ~0.1 cm^{-1} spectral resolution. In the IDIRS (ion-dip infrared spectroscopy) experiments, an additional IR laser beam was used, fired ca. 400 ns prior to the UV lasers. To record the spectra, the IR laser was scanned through the SH-OH stretching mode region, while the excitation laser was kept tuned to an electronic transition of the species of interest. When the IR photons were resonant with a vibrational transition of the species probed by the UV laser, a dip in the ion signal was produced. In this way, the isomer-specific mass-resolved IR spectra of the species in the beam was recorded.

Rotational spectroscopy. The rotational experiment used a chirped-pulse Fourier transform microwave (CP-FTMW) spectrometer[2–4] operating in the frequency range 2-8 GHz. The spectrometer uses a direct-digital design introduced by Pate,[5] implementing fast-passage[6] rotational broadband excitation through the use of microwave chirped pulses. The chirped pulses (typically 1-4 μs length) are produced with an arbitrary waveform generator (25 GSamples/s), which are later amplified and broadcasted into a jet expansion using a ridged waveguide horn antenna. The first experiments used a power amplifier of 25 W, which was later upgraded to 250 W using a travelling-wave tube. The compounds of 2-phenylethanethiol (b.p. 217°C) and 2-phenylethanol (b.p. 220°C) were obtained commercially and used without further purification. The sample was located inside a heating reservoir attached to a solenoid-driven injector. Both compounds were vaporized at moderate temperatures of

50-75°C. The sample vapors were pressurized with an inert carrier gas, using initially neon (2 bar) and later He-Ar 1:1 (2 bar). The gas expanded supersonically when passing through a circular nozzle (ϕ = 0.8 mm) into the evacuated high-vacuum chamber (ultimate pressure 10^{-7} mbar). The jet was oriented perpendicularly to the exciting radiation. Following the chirped-pulse excitation the sample returns to the initial equilibrium state by emission of a free-induced decay. The time-domain transient emission (40 μs) is recorded was a digital oscilloscope (20 MSamples/s), averaged and Fourier transformed to give the frequency-domain spectrum. One to eight chirped pulses are used per gas pulse. The number of averaged spectra in each spectrum was 1 M cycles, at a repetition rate of 5 Hz. The uncertainty of the frequency measurements was estimated to be better than 20 kHz. All frequency components are referenced to a Rb standard.

Computational methods. The structural screening combined molecular mechanics,[7,8] semiempirical methods[9] and manual searches, covering a large dataset of initial structures. All starting geometries were re-optimized with hybrid (B3LYP[10]) and double-hybrid (B2PLYP[11]) density-functional theory methods incorporating D3[12] empirical dispersion corrections (Becke-Johnson[13] damping function). These computational models used the def2-TZVP basis set.[14] One of the isomers of the PET-PET dimer in Table S2 did not converge. Frequency calculations were performed using the harmonic approximation at the same level of theory. The interaction energies were calculated considering the basis set superposition errors (BSSE).[15] All calculations were performed using Gaussian 16, using tight conditions for geometry optimizations and the ultrafine default for integral calculations.[16] The presence of non-covalent interactions was analysed using the NCIplot method,[17,18] based on a reduced gradient of electronic density. The physical contributions to the binding potential of the water clusters were estimated by energy decomposition analysis using second-order symmetry adapted perturbation theory[19] (SAPT), implemented in PSI4.[20]

REFERENCES

(1) León, I.; Lesarri, A.; Fernández, J. A. Evaluation of the Aggregation Process in a Mixture of Propofol and Benzocaine. *Phys. Chem. Chem. Phys.* **2019**, *21* (7), 3537–3544. DOI: 10.1039/c8cp04386h.

(2) Grabow, J.-U. Fourier Transform Microwave Spectroscopy Measurement and Instrumentation. In *Handbook of High-resolution Spectroscopy*; Merkt, F., Quack, M., Eds.; John Wiley & Sons, Ltd: New York, 2011; pp 723–799. DOI: 10.1002/9780470749593.hrs037.

(3) Shipman, S. T.; Pate, B. H. New Techniques in Microwave Spectroscopy. In *Handbook of High-resolution Spectroscopy*; Merkt, F., Quack, M., Eds.; Major Reference Works; John Wiley & Sons, Ltd: New York, 2011; pp 801–828. DOI: 10.1002/9780470749593.hrs036.

(4) Caminati, W.; Grabow, J.-U. Advancements in Microwave Spectroscopy. In *Frontiers and Advances in Molecular Spectroscopy*; Laane, J., Ed.; Elsevier Inc., 2018; pp 569–598. DOI: 10.1016/B978-0-12-811220-5.00018-6.

(5) Neill, J. L.; Shipman, S. T.; Alvarez-Valtierra, L.; Lesarri, A.; Kisiel, Z.; Pate, B. H. Rotational Spectroscopy of Iodobenzene and Iodobenzene-Neon with a Direct Digital 2-8 GHz Chirped-Pulse Fourier Transform Microwave Spectrometer. *J. Mol. Spectrosc.* **2011**, *269* (1), 21–29. DOI: 10.1016/j.jms.2011.04.016.

(6) McGurk, J. C.; Schmalz, T. G.; Flygare, W. H. Fast Passage in Rotational Spectroscopy: Theory and Experiment. *J. Chem. Phys.* **1974**, *60* (11), 4181–4188. DOI: 10.1063/1.1680886.

(7) Halgren, T. A. Merck Molecular Force Field. II. MMFF94 van Der Waals and Electrostatic Parameters for Intermolecular Interactions. *J. Comput. Chem.* **1996**, *17* (5–6), 520–552. DOI: 10.1002/(SICI)1096-987X(199604)17:5/6<520::AID-JCC2>3.0.CO;2-W.

(8) Halgren, T. A. MMFF VI. MMFF94s Option for Energy Minimization Studies. *J. Comput. Chem.* **1999**, *20* (7), 720–729. DOI: 10.1002/(SICI)1096-987X(199905)20:7<720::AID-JCC7>3.0.CO;2-X.

(9) Pracht, P.; Bohle, F.; Grimme, S. Automated Exploration of the Low-Energy Chemical Space with Fast Quantum Chemical Methods. *Phys. Chem. Chem. Phys.* **2020**, *22* (14), 7169–7192. DOI: 10.1039/C9CP06869D.

(10) Becke, A. D. Density-Functional Thermochemistry. III. The Role of Exact Exchange. *J. Chem. Phys.* **1993**, *98* (7), 5648–5652. DOI: 10.1063/1.464913.

(11) Grimme, S.; Neese, F. Double-Hybrid Density Functional Theory for Excited Electronic States of Molecules. *J. Chem. Phys.* **2007**, *127* (15), 1–18. DOI: 10.1063/1.2772854.

(12) Becke, A. D.; Johnson, E. R. A Density-Functional Model of the Dispersion Interaction. *J. Chem. Phys.* **2005**, *123* (15), 154101. DOI: 10.1063/1.2065267.

(13) Grimme, S.; Ehrlich, S.; Goerigk, L. Effect of the Damping Function in Dispersion Corrected Density Functional Theory. *J. Comput. Chem.* **2011**, *32* (7), 1456–1465. DOI: 10.1002/jcc.21759.

(14) Weigend, F.; Ahlrichs, R. Balanced Basis Sets of Split Valence, Triple Zeta Valence and Quadruple Zeta Valence Quality for H to Rn: Design and Assessment of Accuracy. *Phys. Chem. Chem. Phys.* **2005**, *7* (18), 3297. DOI: 10.1039/b508541a.

(15) Boys, S. F.; Bernardi, F. The Calculation of Small Molecular Interactions by the Differences of Separate Total Energies. Some Procedures with Reduced Errors. *Mol. Phys.* **1970**, *19* (4), 553–566. DOI: 10.1080/00268977000101561.

(16) Frisch, M. J.; Trucks, G. W.; Schlegel, H. B.; Scuseria, G. E.; Robb, M. A.; Cheeseman, J. R.; Scalmani, G.; Barone, V.; Petersson, G. A.; Nakatsuji, H.; Li, X.; Caricato, M.; Marenich, A. V.; Bloino, J.; Janesko, B. G.; Gomperts, R.; Mennucci, B.; Hratchian, H. P.; Ortiz, J. V.; Izmaylov, A. F.; Sonnenberg, J. L.; Williams; Ding, F.; Lipparini, F.; Egidi, F.; Goings, J.; Peng, B.; Petrone, A.; Henderson, T.; Ranasinghe, D.; Zakrzewski, V. G.; Gao, J.; Rega, N.; Zheng, G.; Liang, W.; Hada, M.; Ehara, M.; Toyota, K.; Fukuda, R.; Hasegawa, J.; Ishida, M.; Nakajima, T.; Honda, Y.; Kitao, O.; Nakai, H.; Vreven, T.; Throssell, K.; Montgomery Jr., J. A.; Peralta, J. E.; Ogliaro, F.; Bearpark, M. J.; Heyd, J. J.; Brothers, E. N.; Kudin, K. N.; Staroverov, V. N.; Keith, T. A.; Kobayashi, R.; Normand, J.; Raghavachari, K.; Rendell, A. P.; Burant, J. C.; Iyengar, S. S.; Tomasi, J.; Cossi, M.; Millam, J. M.; Klene, M.; Adamo, C.; Cammi, R.; Ochterski, J. W.; Martin, R. L.; Morokuma, K.; Farkas, O.; Foresman, J. B.; Fox, D. J. Gaussian 16, Rev. C.01. Gaussian, Inc: Wallingford CT 2016.

(17) Johnson, E. R.; Keinan, S.; Mori-Sánchez, P.; Contreras-García, J.; Cohen, A. J.; Yang, W. Revealing Noncovalent Interactions. *J. Am. Chem. Soc.* **2010**, *132* (18), 6498–6506. DOI: 10.1021/ja100936w.

(18) Contreras-García, J.; Johnson, E. R.; Keinan, S.; Chaudret, R.; Piquemal, J. P.; Beratan, D. N.; Yang, W. NCIPLOT: A Program for Plotting Noncovalent Interaction Regions. *J. Chem. Theory Comput.* **2011**, *7* (3), 625–632. DOI: 10.1021/ct100641a.

(19) Jeziorski, B.; Moszynski, R.; Szalewicz, K. Perturbation Theory Approach to Intermolecular Potential Energy Surfaces of van Der Waals Complexes. *Chem. Rev.* **1994**, *94* (7), 1887–1930. DOI: 10.1021/cr00031a008.

(20) Parrish, R. M.; Burns, L. A.; Smith, D. G. A.; Simmonett, A. C.; DePrince, A. E.; Hohenstein, E. G.; Bozkaya, U.; Sokolov, A. Y.; Di Remigio, R.; Richard, R. M.; Gonthier, J. F.; James, A. M.; McAlexander, H. R.; Kumar, A.; Saitow, M.; Wang, X.; Pritchard, B. P.; Verma, P.; Schaefer, H. F.; Patkowski, K.; King, R. A.; Valeev, E. F.; Evangelista, F. A.; Turney, J. M.; Crawford, T. D.; Sherrill, C. D. Psi4 1.1: An Open-Source Electronic Structure Program Emphasizing Automation, Advanced Libraries, and Interoperability. *J. Chem. Theory Comput.* **2017**, *13* (7), 3185–3197. DOI: 10.1021/acs.jctc.7b00174.

Figure S1. The two observed conformations of the monomers of 2-phenylethanethiol (upper row) and 2-phenylethanol (lower row). The global minimum of the two molecules is conformer Ggπ (gauche-gauche), orienting the polar groups to the π ring. However, the second most stable conformer is Ag (antiperiplanar-gauche) in 2-phenylethanetiol but (C_s) plane-symmetric At (anti-anti) in 2-phenylethanol. Each conformer (except the symmetric At) has two equivalent enantiomeric species. The relative energies (in kJ mol^{-1}) in the drawing were calculated at the B3LYP-D3(BJ)/def2-TZVP level.

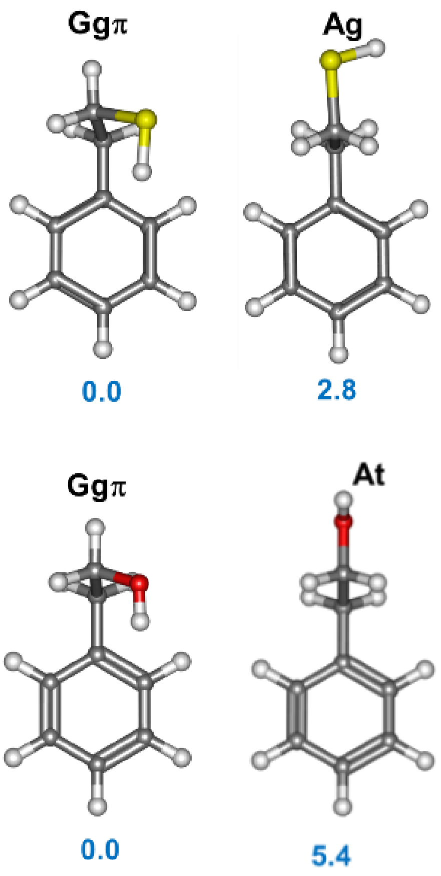

Figure S2. Transient enantiomeric pairs in 2-phenylethanethiol and notation for the most stable gauche-gauche molecular conformation (Ggπ).

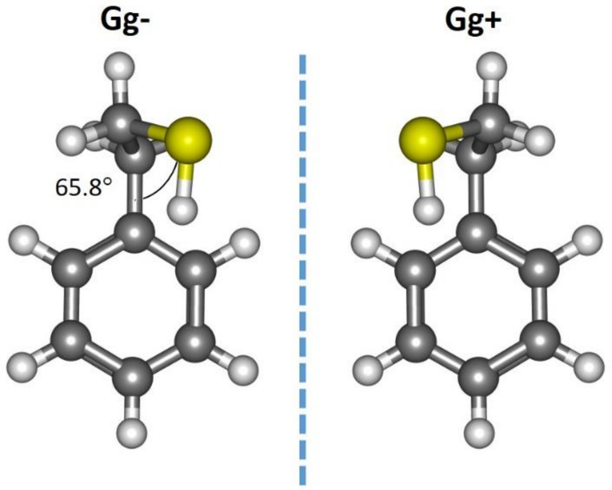

Figure S3. The three observed lowest-energy conformations of the 2-phenylethanol dimer (PEAL-PEAL), previously reported (*Phys. Chem. Chem. Phys.* **2022**, *24*, 24800). Calculations were performed at the B3LYP-D3(BJ)/def2-TZVP level.

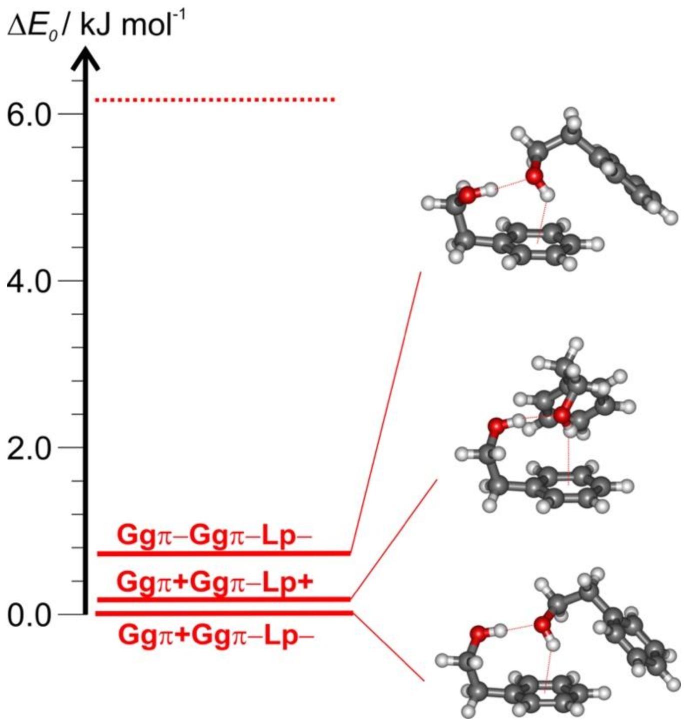

Figure S4. Jet-cooled electronic REMPI spectra of 2-phenylethanethiol (PET), 2-phenylethanol (PEAL) and the dimers of PET-PET and PET-PEAL.

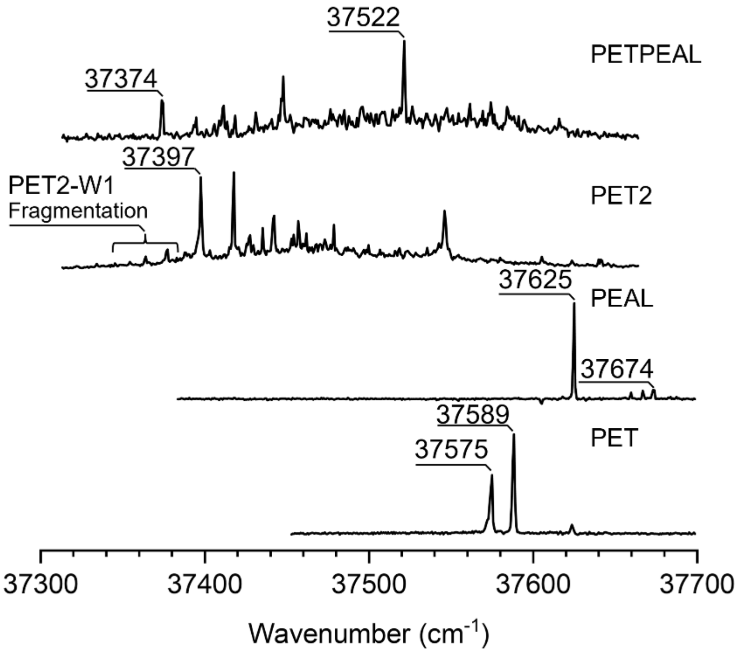

Figure S5. (Upper panel) Comparison between the 2c-REMPI spectrum of 2-phenylethanol and the UV/UV hole burning traces obtained probing the bands at 37627 cm^{-1} (Isomer Ggπ) and 37675 cm^{-1} (Isomer At). (Lower panel) Similar comparison for 2-phenylethanethiol.

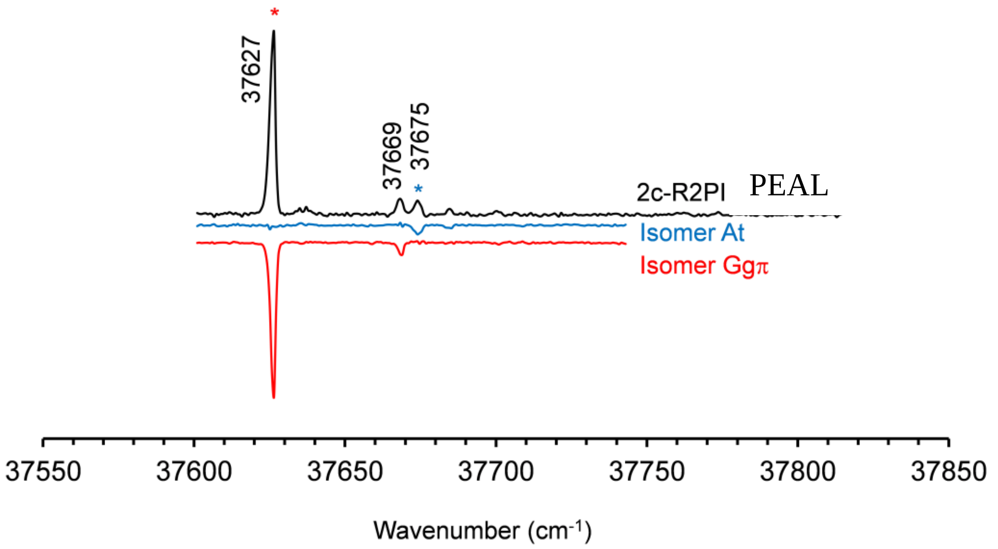

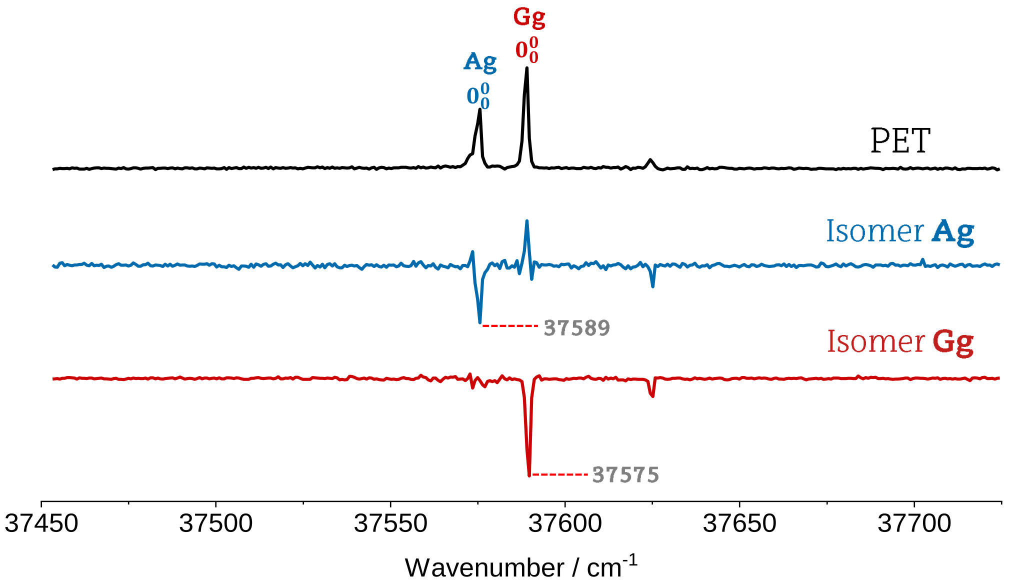

Figure S6. (Upper panel) Comparison between the IDIRS spectra of 2-phenylethanol obtained probing the same UV transitions at 37627 cm^{-1} (Isomer Ggπ) and 37675 cm^{-1} (Isomer At) as in the hole burning and the simulations obtained using normal mode analysis (red traces) and anharmonic calculations (blue trace). A correction factor of 0.963 was used for the harmonic simulation. (Lower panel) Similar comparison for the most abundant isomer of 2-phenylethanethiol, probing the UV transition at 37589 cm^{-1} (Isomer Ggπ). As can be seen, the S-H stretching band is too weak to be detected and therefore, the IDIR spectrum of the weaker isomer was not measured. Calculations were performed at the B3LYP-D3(BJ)/def2-TZVP level.

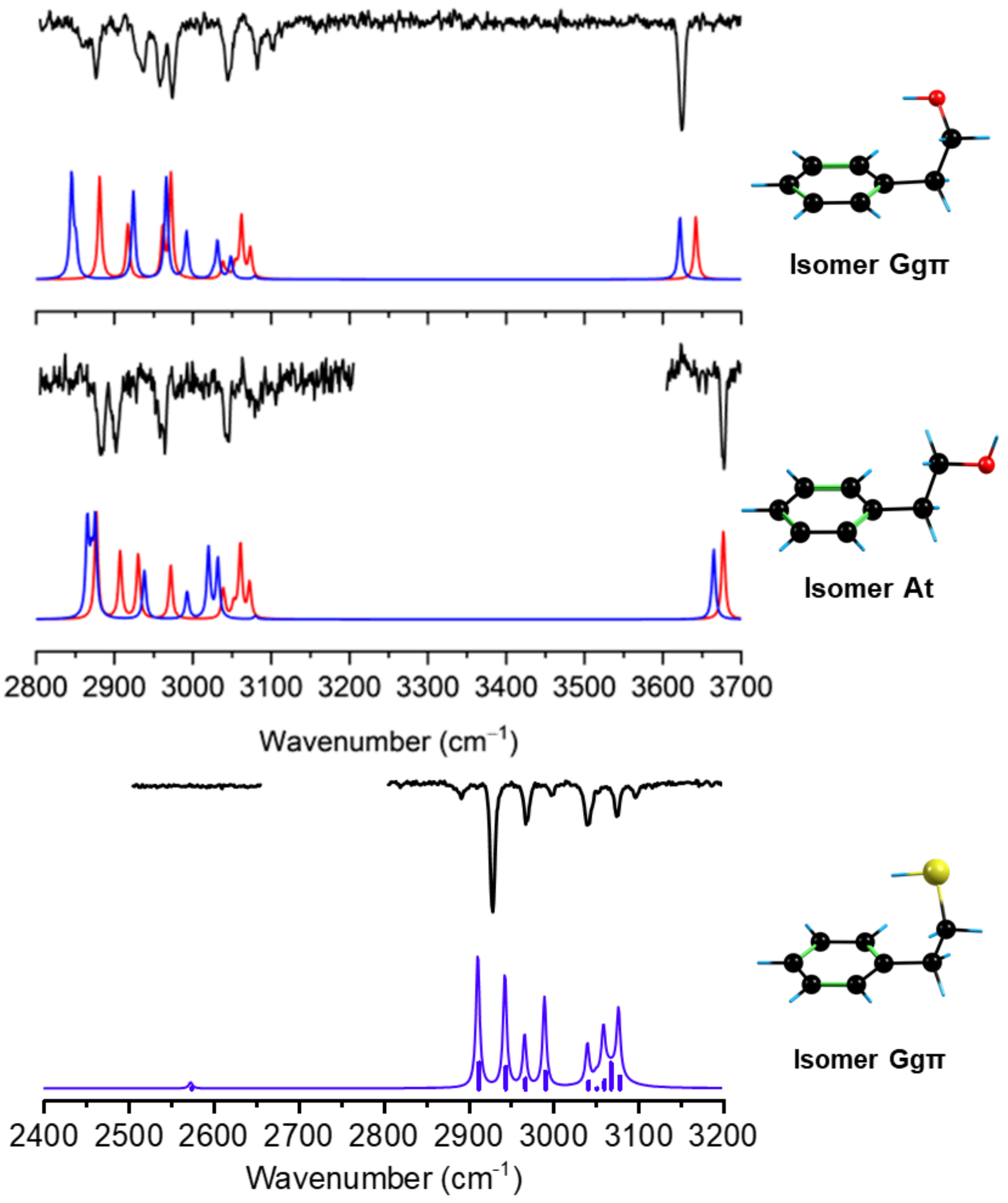

Figure S7. The most stable isomers of the 2-phenylethanethiol homodimer (PET-PET), classified by their intermolecular interactions and ordered by electronic energy (ΔE, kJ/mol). Isomers in the orange and green rectangles exhibit a thiol-thiol S-H···S hydrogen bond. Calculations were performed at the B2PLYP-D3(BJ)/def2-TZVP level.

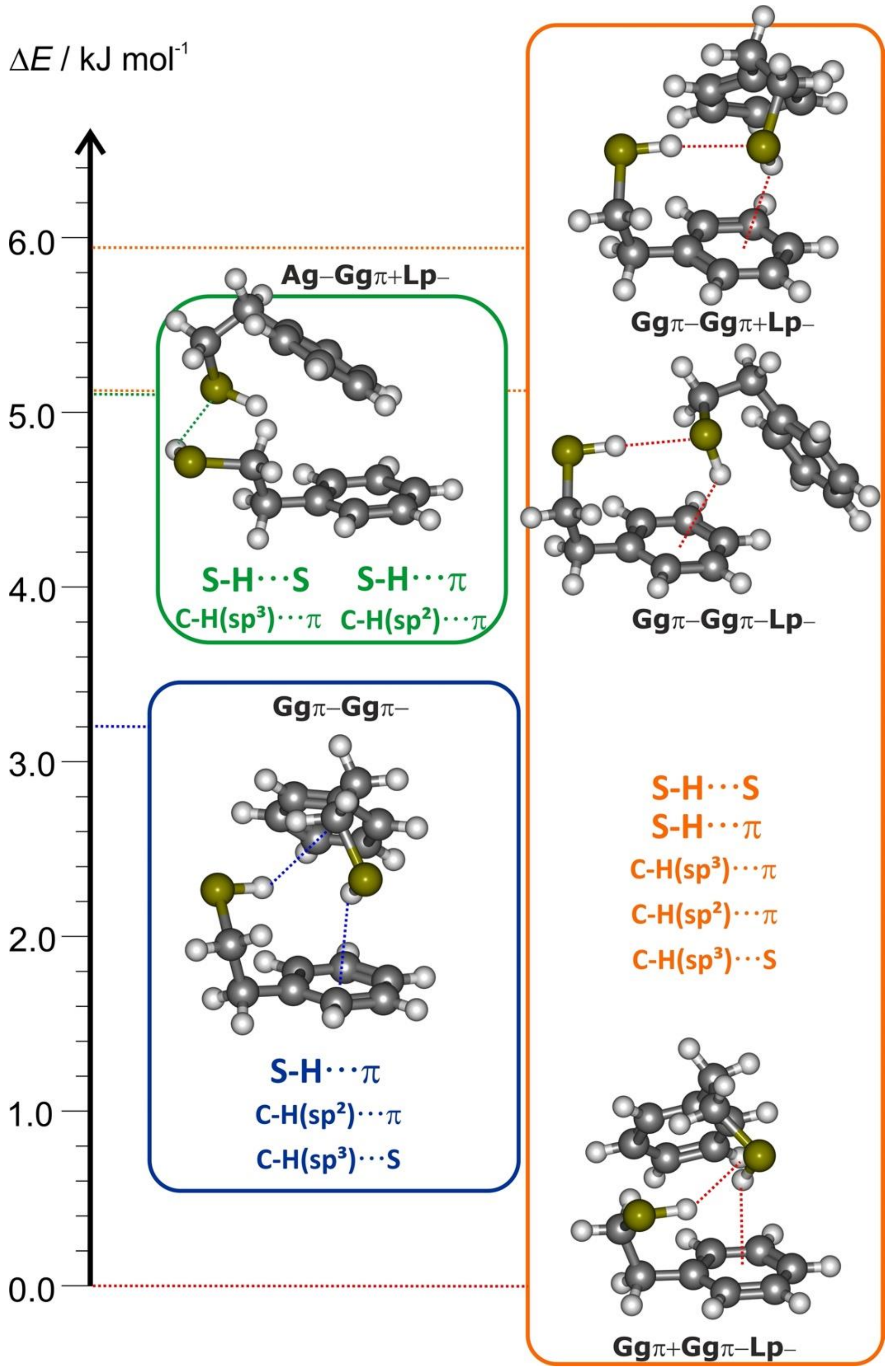

Figure S8. The most stable isomers of the 2-phenylethanethiol···2-phenylethanol dimer (PET-PEAL), classified by their intermolecular interactions and ordered by electronic energy (Δ*E*, kJ/mol). Calculations were performed at the B2PLYP-D3(BJ)/def2-TZVP level.

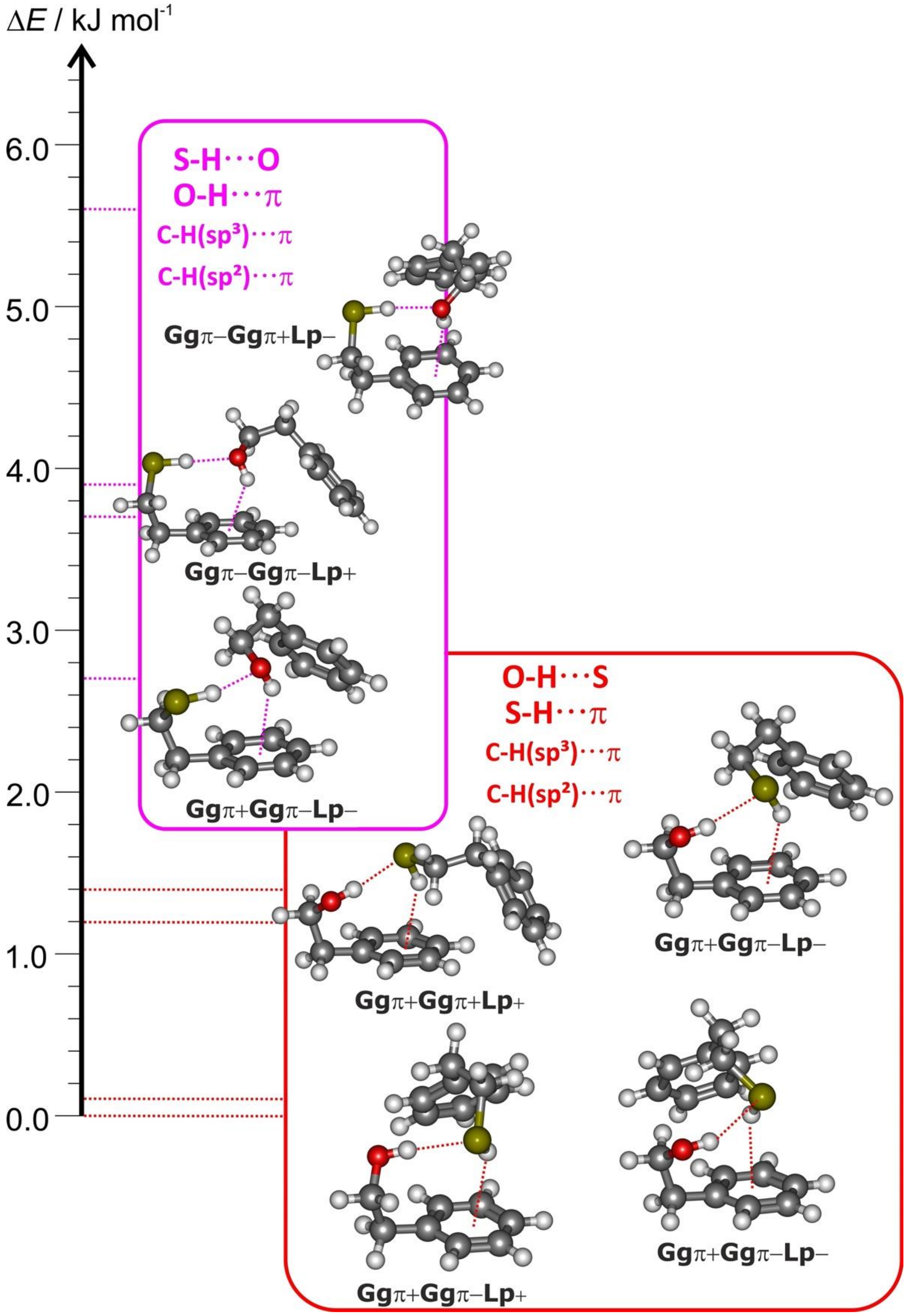

Figure S9. Comparison between IDIR spectrum obtained probing the UV transition at 37418 cm^{-1} and B3LYP-D3(BJ)/ def2-TZVP predictions for the dimer of 2-phenylethanethiol (PET-PET).

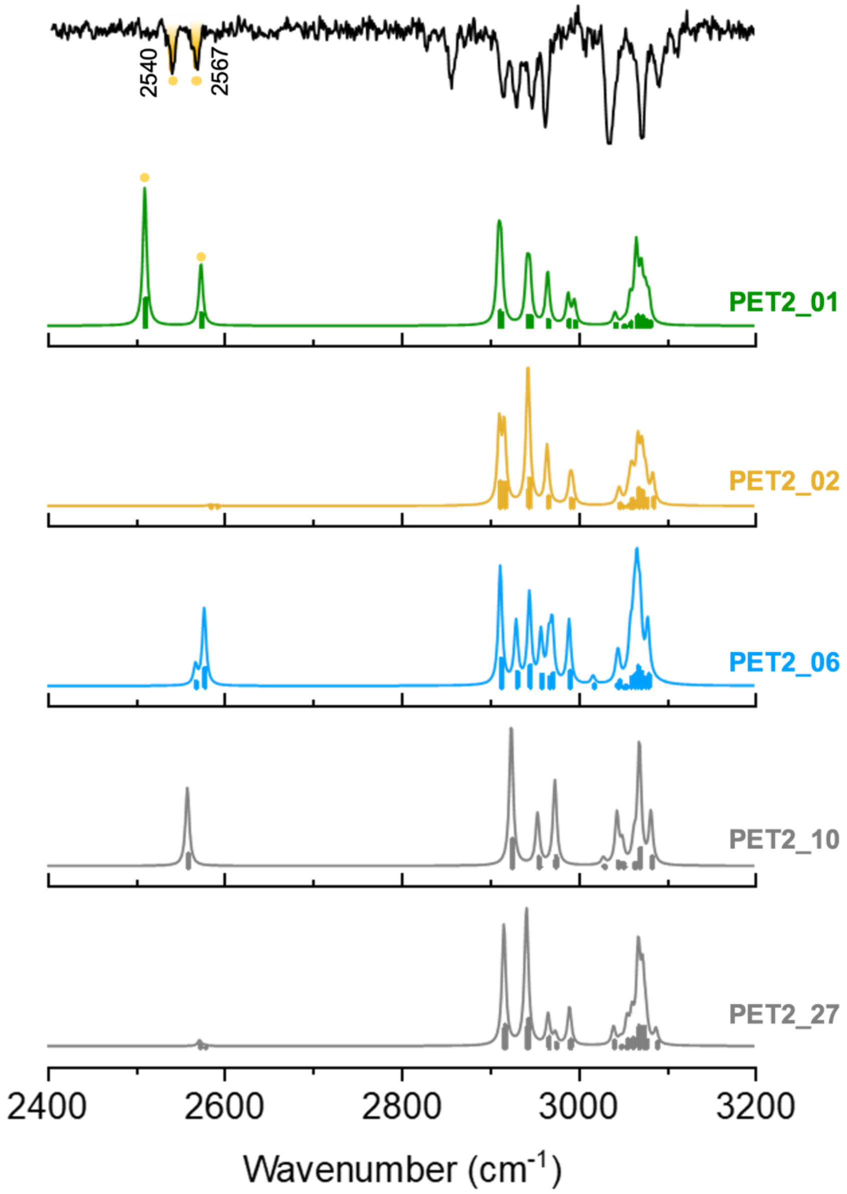

Figure S10. IR-UV hole burning spectrum of the heterodimer between 2-pheneylethanethiol and 2-phenylethanol (PET-PEAL), obtained probing the IR transitions at 3499 cm^{-1} (Isomer 1) and 3484 cm^{-1} (Isomer 2), confirming the detection of two isomers of the aggregate.

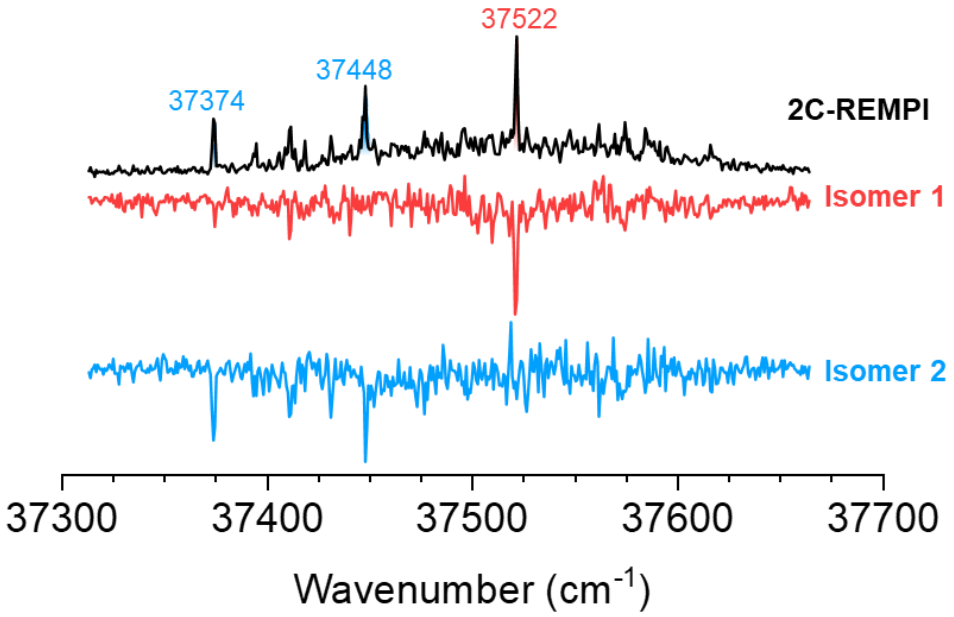

Figure S11. Comparison between IDIR spectra of the heterodimers between 2-pheneylethanethiol and 2-phenylethanol (PET-PEAL) obtained probing the UV transitions at 37522 cm^{-1} (Isomer 1) and 37448 cm^{-1} (Isomer 2) and the predictions at the B3LYP-D3(BJ)/ def2-TZVP computational level.

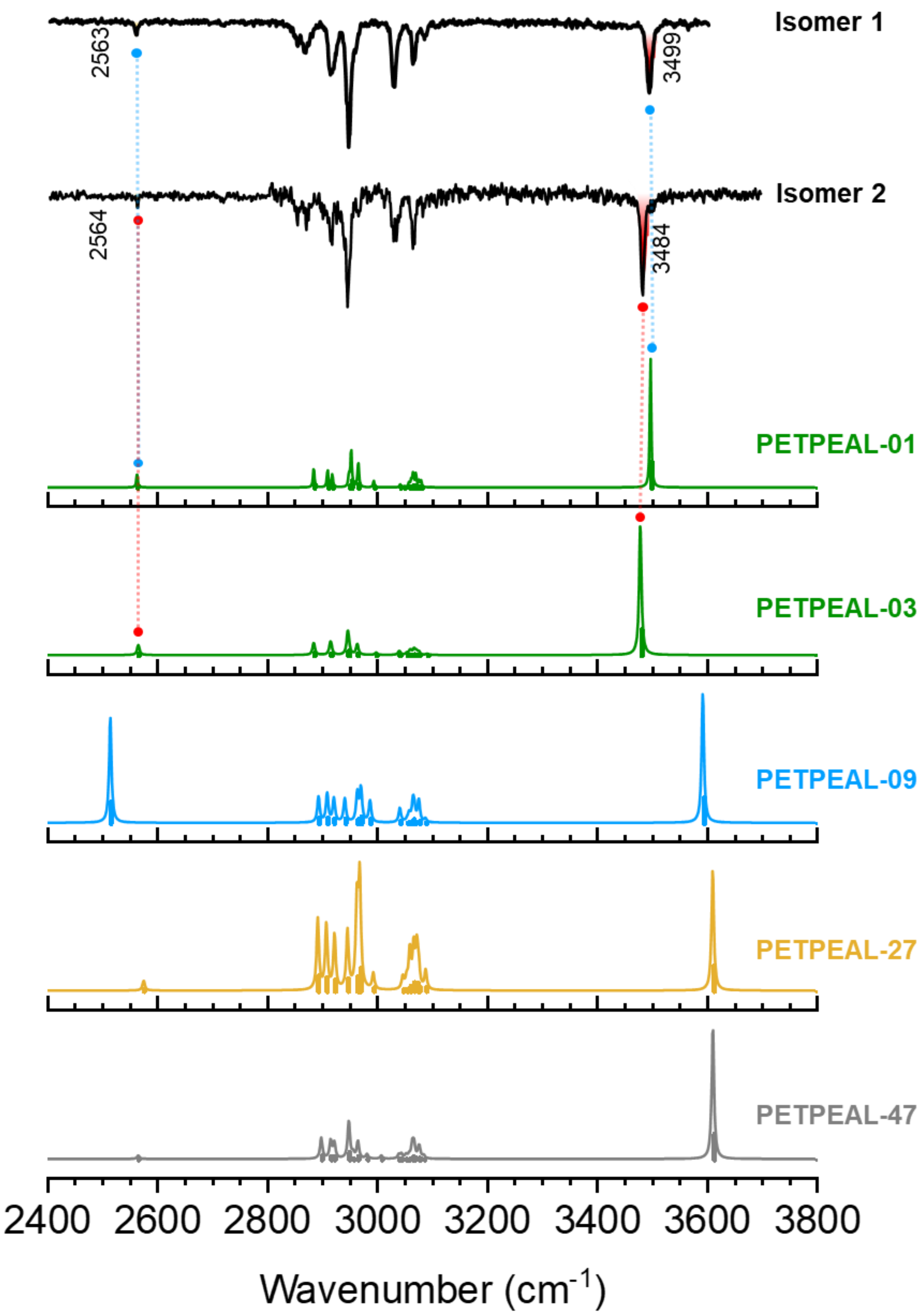

Figure S12. The microwave spectrum of a co-expansion of 2-phenylethanethiol (PET) and 2-phenylethanol (PEAL) in the 2-8 GHz frequency region (upper panel), and a section of the rotational spectrum illustrating the assignment of PET-PEAL (lower panel). The positive trace shows the experimental spectrum. The negative trace is the simulation using the fitted rotational constants for isomer 2 in Tables 1 and S3-S4.

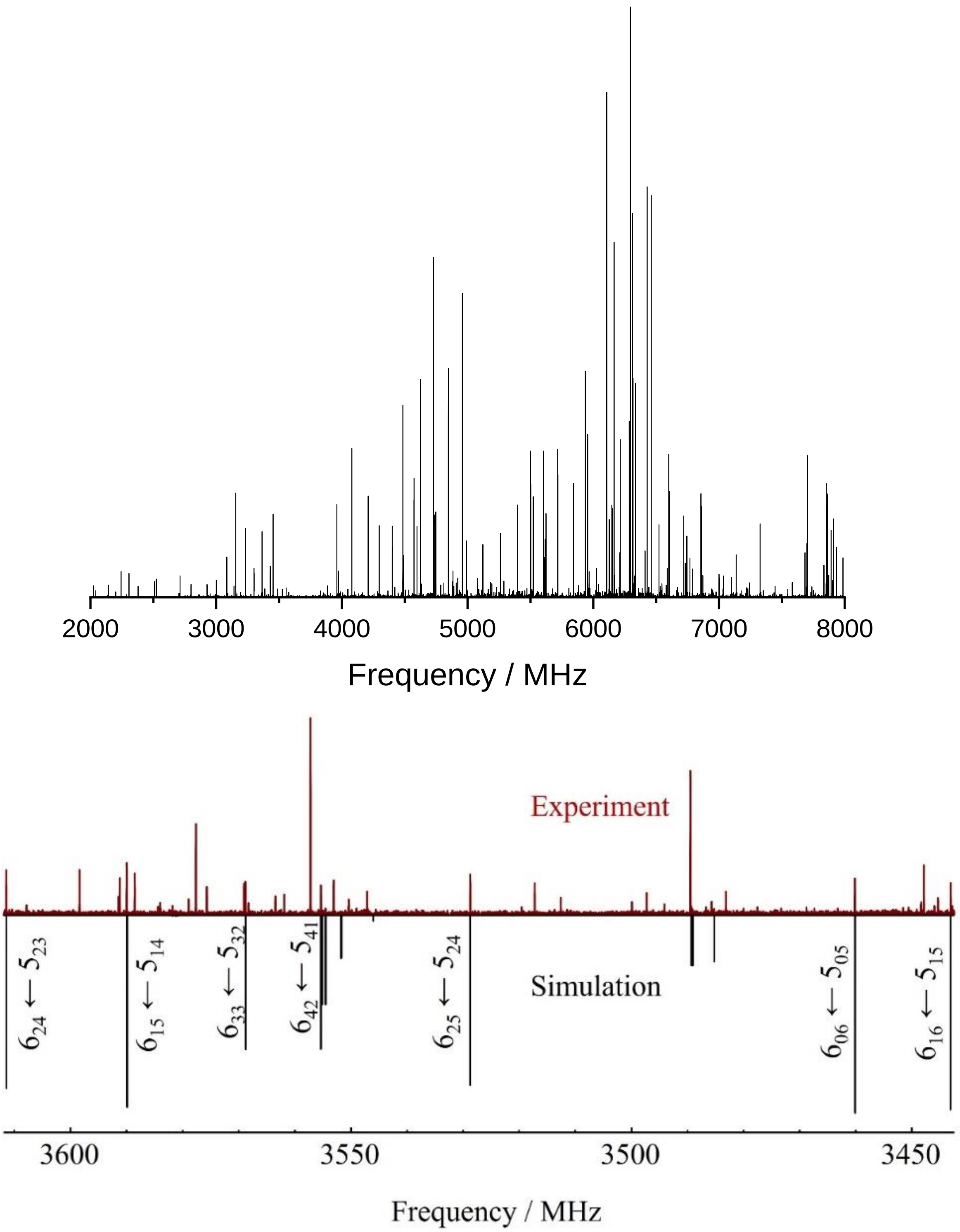

Figure S13. A rotatable 3D figure of the predicted global minimum (isomer 1) of the 2-phenylethanethiol dimer (PET-PET).

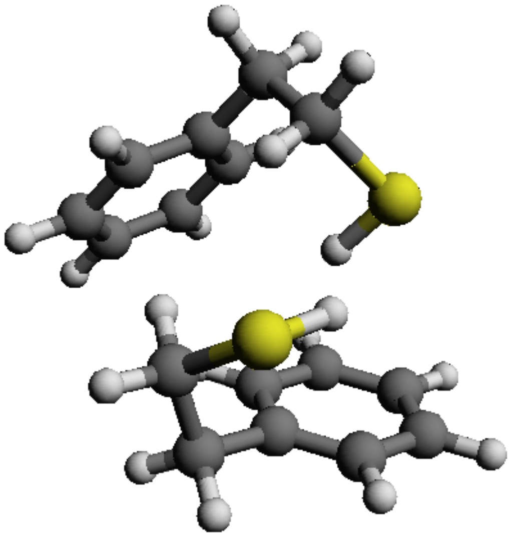

Figure S14. A rotatable 3D figure of the predicted global minimum (isomer 1) of the dimer of 2-phenylethanethiol and 2-phenylethanol (PET-PEAL).

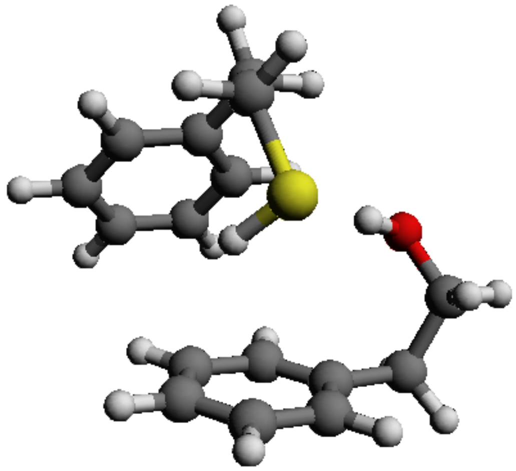

Figure S15. A rotatable 3D figure of the second predicted species (isomer 2) of the dimer of 2-phenylethanethiol and 2-phenylethanol (PET-PEAL).

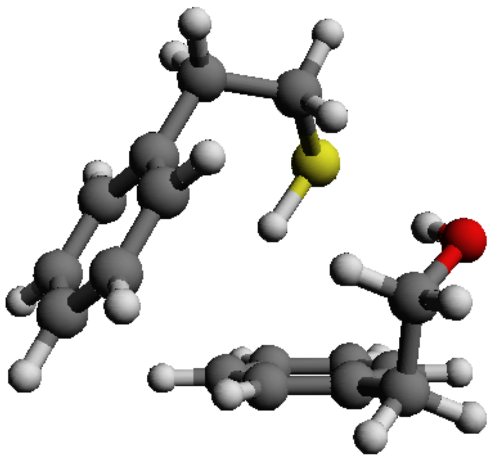

Figure S16. A rotatable 3D figure of the third predicted species (isomer 3) of the dimer of 2-phenylethanethiol and 2-phenylethanol (PET-PEAL).

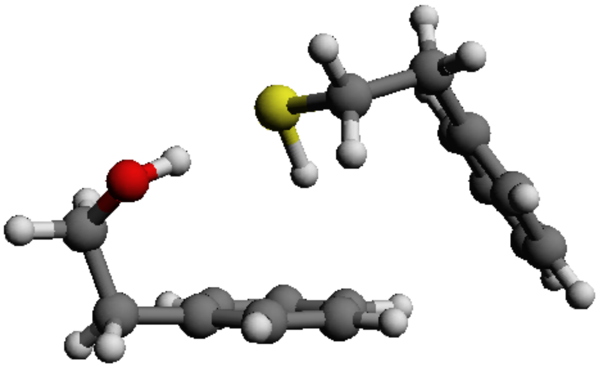

Table S1. Conformational search for the homodimer of 2-phenylethanethiol (PET-PET) using the B3LYP-D3(BJ) method (tight convergence, ultrafine integration) and the def2-TZVP basis set, and comparison with the experimental rotational parameters.

	Experiment	Isomer 1	Isomer 2	Isomer 3
		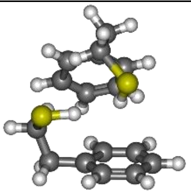 Ggπ+Ggπ–Lp–	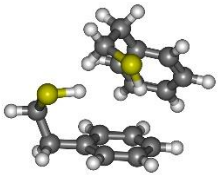 Ggπ+Ggπ–Lp–	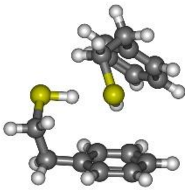 Ggπ–Ggπ–
Rotational parameters				
A / MHz [a]	390.86720(30) [e]	395.0	482.8	403.5
B / MHz	279.72785(19)	288.2	244.1	289.3
C / MHz	262.05574(21)	266.9	213.9	269.5
D_J / kHz	0.02677(61)	0.0174	0.0323	0.0242
D_{JK} / kHz	0.1007(20)	0.0759	-0.1542	0.1199
D_K / kHz	-0.1020(32)	-0.0774	0.4044	-0.1138
d_1 / kHz	0.00434(49)	0.0018	-0.0029	-0.0032
d_2 / kHz	-0.00142(22)	-0.0020	-0.0002	-0.0003
$\|\mu_a\|$ / D	+++	1.8	1.7	0.0
$\|\mu_b\|$ / D	+	0.9	0.9	0.2
$\|\mu_c\|$ / D	+	1.1	1.8	0.1
μ_{TOTAL} / D		2.3	2.6	0.2
Structural parameters				
HBond donor:				
HS–CβCα / deg [b]		67.4	66.9	-61.1
SCβ–CαC1 / deg		-69.5	-67.3	71.2
HBond acceptor:				
HS–CβCα / deg		-59.70	-74.4	-58.7
SCβ–CαC1 / deg		67.0	69.5	65.3
r(H···S) / Å		2.669	2.621	-
∠(S–H···S) / deg		151.1	157.1	-
r(S–H···centroid) / Å		2.591	2.625	2.686/3.168
Energetic parameters				
ΔE/ kJ mol^{-1} [c]		0.0	3.9	3.9
ΔG / kJ mol^{-1}		0.0	2.2	1.0
Ec / kJ mol^{-1}		-41.9	-38.6	-37.7
ΔEc / kJ mol^{-1}		0.0	3.3	4.2
N [d]	289			
σ / kHz	11.6			

[a]Rotational constants (A, B, C), Watson's S-reduction centrifugal distortion constants (D_J, D_{JK}, D_K, d_1, d_2) and electric dipole moments (μ_α, α = a, b, c). [b]Structural parameters of the dimer. [c]Relative electronic energies (ΔE) with zero-point correction, Gibbs energy (ΔG, 298K, 1 atm), complexation energies (E_c) and relative complexation energies (ΔE_c). [d]Number of transitions (N) and rms deviation (σ) of the fit. [e]Standard errors in units of the last digit.

Table S1. Continued.

	Isomer 4	Isomer 5	Isomer 6
	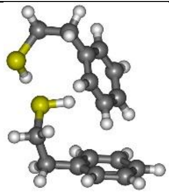	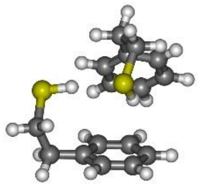	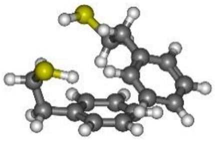
	Ggπ–Ggπ–Lp–	Ggπ–Ggπ+Lp–	Ag–Ggπ+ Lp–
Rotational parameters			
A / MHz[a]	562.2	418.5	415.0
B / MHz	219.9	271.2	270.8
C / MHz	186.1	251.0	241.8
D_J / kHz	0.0114	0.0237	0.0195
D_{JK} / kHz	-0.0127	-0.0071	-0.0211
D_K / kHz	0.1019	0.0112	0.0534
d_1 / kHz	-0.0016	-0.0012	-0.0008
d_2 / kHz	-0.0001	-0.0005	-0.0006
$\|\mu_a\|$ / D	1.3	0.7	0.6
$\|\mu_b\|$ / D	1.3	0.8	1.8
$\|\mu_c\|$ / D	0.2	0.5	0.2
μ_{TOTAL} / D	1.8	1.2	1.9
Structural parameters			
HBond donor:			
HS–CβCα / deg [b]	-79.9	-78.4	-62.8
SCβ–CαC1 / deg	66.2	67.4	173.0
HBond acceptor:			
HS–CβCα / deg	-77.0	62.0	51.7
SCβ–CαC1 / deg	67.7	-65.0	-71.2
r(H···S) / Å	2.557	2.565	2.923
∠(S–H···S) / deg	173.84	167.8	134.4
r(S–H···centroid) / Å	2.613	2.634	4.062
Energetic parameters			
ΔE/ kJ mol^{-1}[c]	5.5	5.6	5.9
ΔG / kJ mol^{-1}	4.3	3.3	6.0
Ec / kJ mol^{-1}	-38.9	-39.3	-41.8
ΔEc / kJ mol^{-1}	3.0	2.6	0.1

[a]Rotational constants (A, B, C), Watson's S-reduction centrifugal distortion constants (D_J, D_{JK}, D_K, d_1, d_2) and electric dipole moments (μ_α, α = a, b, c). [b]Structural parameters of the dimer. [c]Relative electronic energies (ΔE) with zero-point correction, Gibbs energy (ΔG, 298K, 1 atm), complexation energies (E_c) and relative complexation energies (ΔE_c). [d]Number of transitions (N) and rms deviation (σ) of the fit. [e]Standard errors in units of the last digit.

Table S1. Continued.

	Isomer 7	Isomer 8	Isomer 9
	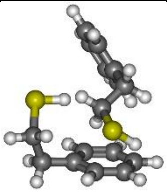	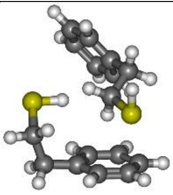	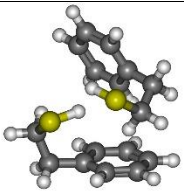
	Ggπ–Ag–	Ggπ–Ag+	Ggπ+Ggπ+
Rotational parameters			
A / MHz[a]	350.4	388.5	408.9
B / MHz	296.1	283.9	266.6
C / MHz	250.9	241.4	256.6
D_J / kHz	0.0427	0.0281	0.0205
D_{JK} / kHz	-0.0013	-0.0422	0.0100
D_K / kHz	-0.0263	0.1065	-0.0025
d_1 / kHz	-0.0108	0.0018	-0.0050
d_2 / kHz	0.0017	-0.0008	-0.0010
$\|\mu_a\|$ / D	0.6	0.0	2.2
$\|\mu_b\|$ / D	0.8	0.1	0.6
$\|\mu_c\|$ / D	1.0	0.0	1.8
μ_{TOTAL} / D	1.4	0.1	2.9
Structural parameters			
HBond donor:			
HS–CβCα / deg [b]	-63.7	-64.5	63.2
SCβ–CαC1 / deg	68.2	72.1	-69.9
HBond acceptor:			
HS–CβCα / deg	-64.0	64.5	77.5
SCβ–CαC1 / deg	176.8	175.0	-67.0
r(H···S) / Å	-	-	2.945
∠(S–H···S) / deg	-	-	128.4
r(S–H···centroid) / Å	2.653	2.653	2.697
Energetic parameters			
ΔE/ kJ mol^{-1}[c]	7.4	7.7	7.8
ΔG / kJ mol^{-1}	2.7	2.8	5.9
*E*c / kJ mol^{-1}	-37.3	-37.6	-35.2
ΔEc / kJ mol^{-1}	4.6	4.3	6.7

[a]Rotational constants (*A*, *B*, *C*), Watson's S-reduction centrifugal distortion constants (D_J, D_{JK}, D_K, d_1, d_2) and electric dipole moments (μ_α, α = a, b, c). [b]Structural parameters of the dimer. [c]Relative electronic energies (ΔE) with zero-point correction, Gibbs energy (ΔG, 298K, 1 atm), complexation energies (E_c) and relative complexation energies (ΔE_c). [d]Number of transitions (*N*) and rms deviation (σ) of the fit. [e]Standard errors in units of the last digit.

Table S2. Conformational search for the homodimer of 2-phenylethanethiol (PET-PET) using the B2PLYP-D3(BJ) method (tight convergence, ultrafine integration) and the def2-TZVP basis set, and comparison with the experimental rotational parameters.

	Experiment	Isomer 1	Isomer 2	Isomer 3
		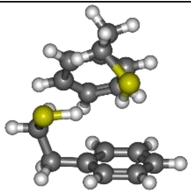	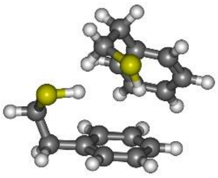	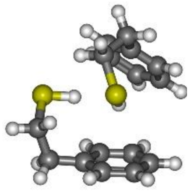
		Ggπ+Ggπ–Lp–	Ggπ+Ggπ–Lp–	Ggπ–Ggπ–
Rotational parameters				
A / MHz [a]	390.86720(30) [e]	394.4	Not	403.7
B / MHz	279.72785(19)	286.3	converged	287.5
C / MHz	262.05574(21)	266.8		268.8
D_J / kHz	0.02677(61)	0.0189		0.0242
D_{JK} / kHz	0.1007(20)	0.0803		0.1456
D_K / kHz	-0.1020(32)	-0.0817		-0.1407
d_1 / kHz	0.00434(49)	0.0024		-0.0025
d_2 / kHz	-0.00142(22)	-0.0021		-0.0008
$\|\mu_a\|$ / D	+++	1.7		0.0
$\|\mu_b\|$ / D	+	0.9		0.2
$\|\mu_c\|$ / D	+	1.1		0.1
μ_{TOTAL} / D				0.2
Structural parameters				
HBond donor:				
HS–CβCα / deg [b]		66.4		-61.0
SCβ–CαC1 / deg		-69.3		70.8
HBond acceptor:				
HS–CβCα / deg		-60.6		-58.8
SCβ–CαC1 / deg		66.8		65.2
r(H···S) / Å		2.714		-
∠(S–H···S) / deg		150.9		-
r(S–H···centroid) / Å		2.600		2.699/3.194
Energetic parameters				
ΔE/ kJ mol^{-1} [c]		0.0		3.4
ΔG / kJ mol^{-1}		0.0		0.9
*E*c / kJ mol^{-1}		-35.4		-31.9
ΔEc / kJ mol^{-1}		0.1		3.6
N [d]	289			
σ / kHz	11.6			

[a]Rotational constants (*A, B, C*), Watson's S-reduction centrifugal distortion constants (D_J, D_{JK}, D_K, d_1, d_2) and electric dipole moments (μ_α, α = a, b, c). [b]Structural parameters of the dimer. [c]Relative electronic energies (Δ*E*) with zero-point correction, Gibbs energy (Δ*G*, 298K, 1 atm), complexation energies (E_c) and relative complexation energies (ΔE_c). [d]Number of transitions (*N*) and rms deviation (σ) of the fit. [e]Standard errors in units of the last digit.

Table S2. Continued.

	Isomer 4	Isomer 5	Isomer 6
	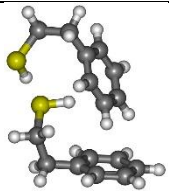	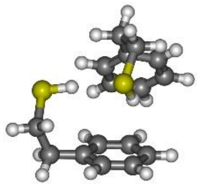	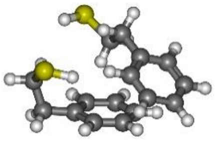
	Ggπ–Ggπ–Lp–	Ggπ–Ggπ+Lp–	Ag–Ggπ+ Lp–
<u>Rotational parameters</u>			
A / MHz[a]	562.1	418.3	413.0
B / MHz	218.6	268.9	270.3
C / MHz	185.2	250.6	242.0
D_J / kHz	0.0137	0.0269	0.0203
D_{JK} / kHz	-0.0221	-0.0023	-0.0240
D_K / kHz	0.1231	0.0044	0.0590
d_1 / kHz	-0.0018	-0.0013	-0.0009
d_2 / kHz	-0.0001	-0.0006	-0.0006
$\|\mu_a\|$ / D	1.3	0.8	0.6
$\|\mu_b\|$ / D	1.3	0.9	1.8
$\|\mu_c\|$ / D	0.2	0.5	0.2
μ_{TOTAL} / D	1.8	1.3	1.9
<u>Structural parameters</u>			
HBond donor:			
HS–CβCα / deg [b]	-79.1	-77.7	-62.7
SCβ–CαC1 / deg	66.4	67.5	173.5
HBond acceptor:			
HS–CβCα / deg	-76.6	63.2	52.1
SCβ–CαC1 / deg	67.7	-64.8	-71.1
r(H···S) / Å	2.603	2.620	2.971
∠(S–H···S) / deg	172.2	165.7	134.0
r(S–H···centroid) / Å	2.630	2.622	4.058
<u>Energetic parameters</u>			
ΔE/ kJ mol^{-1}[c]	5.3	5.9	5.3
ΔG / kJ mol^{-1}	4.0	3.2	1.2
Ec / kJ mol^{-1}	-32.9	-35.5	-32.7
ΔEc / kJ mol^{-1}	2.6	0.0	2.8

[a]Rotational constants (A, B, C), Watson's S-reduction centrifugal distortion constants (D_J, D_{JK}, D_K, d_1, d_2) and electric dipole moments (μ_α, α = a, b, c). [b]Structural parameters of the dimer. [c]Relative electronic energies (ΔE) with zero-point correction, Gibbs energy (ΔG, 298K, 1 atm), complexation energies (E_c) and relative complexation energies (ΔE_c). [d]Number of transitions (N) and rms deviation (σ) of the fit. [e]Standard errors in units of the last digit.

Table S2. Continued.

	Isomer 7	Isomer 8	Isomer 9
	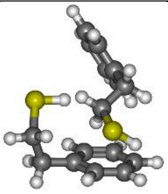	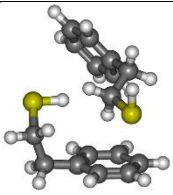	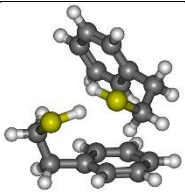
	Ggπ–Ag–	Ggπ–Ag+	Ggπ+Ggπ+
Rotational parameters			
A / MHz[a]	347.8	388.1	408.6
B / MHz	296.8	284.7	265.7
C / MHz	250.2	239.3	255.8
D_J / kHz	0.0563	0.0277	0.0216
D_{JK} / kHz	0.0047	-0.0335	0.0115
D_K / kHz	-0.0358	0.0886	-0.0049
d_1 / kHz	-0.0181	0.0022	-0.0052
d_2 / kHz	-0.0132	-0.0021	-0.0011
$\|\mu_a\|$ / D	0.7	0.0	2.2
$\|\mu_b\|$ / D	0.8	0.1	0.6
$\|\mu_c\|$ / D	1.0	0.0	1.8
μ_{TOTAL} / D	1.5	0.1	2.9
Structural parameters			
HBond donor:			
HS–CβCα / deg [b]	-64.0	-64.8	76.8
SCβ–CαC1 / deg	68.2	72.2	-67.1
HBond acceptor:			
HS–CβCα / deg	-73.7	64.1	63.3
SCβ–CαC1 / deg	177.2	175.6	-69.8
r(H···S) / Å	-	-	2.959
∠(S–H···S) / deg	-	-	102.0
r(S–H···centroid) / Å	2.645	2.700	2.713
Energetic parameters			
ΔE/ kJ mol^{-1}[c]	6.2	6.5	7.1
ΔG / kJ mol^{-1}	1.2	2.3	5.5
E_c / kJ mol^{-1}	-31.7	-32.3	-29.5
ΔE_c / kJ mol^{-1}	3.9	3.3	6.0

[a]Rotational constants (*A*, *B*, *C*), Watson's S-reduction centrifugal distortion constants (D_J, D_{JK}, D_K, d_1, d_2) and electric dipole moments (μ_α, α = a, b, c). [b]Structural parameters of the dimer. [c]Relative electronic energies (Δ*E*) with zero-point correction, Gibbs energy (Δ*G*, 298K, 1 atm), complexation energies (E_c) and relative complexation energies (ΔE_c). [d]Number of transitions (*N*) and rms deviation (*σ*) of the fit. [e]Standard errors in units of the last digit.

Table S3. Conformational search for the heterodimer of 2-phenylethanethiol and 2-phenylethanol (PET-PEAL) using the B3LYP-D3(BJ) method (tight convergence, ultrafine integration) and the def2-TZVP basis set, and comparison with the experimental rotational parameters.

	Experiment	Isomer 1	Isomer 2	Isomer 3
		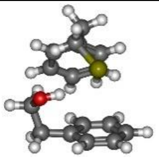 Ggπ+Ggπ−Lp+	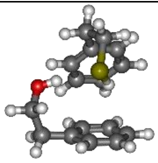 Ggπ+Ggπ−Lp−	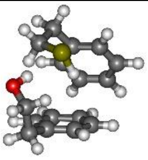 Ggπ+Ggπ+Lp+
Rotational parameters				
A / MHz [a]	456.19818(99) [e]	475.3	459.5	616.2
B / MHz	308.65432(26)	305.8	316.8	256.5
C / MHz	281.92839(29)	279.8	287.7	214.5
D_J / kHz	0.0256(10)	0.0385	0.0219	0.0111
D_{JK} / kHz	0.1077(51)	−0.0518	0.1019	−0.0012
D_K / kHz		0.0663	-0.1049	0.1133
d_1 / kHz		0.0017	-0.0004	-0.0022
d_2 / kHz		-0.0008	-0.0008	-0.0002
$\|\mu_a\|$ / D	++	0.8	2.2	1.9
$\|\mu_b\|$ / D	+	0.2	1.2	1.0
$\|\mu_c\|$ / D		1.2	0.1	0.0
μ_{TOTAL} / D		1.4	2.5	2.1
Structural parameters				
HBond donor				
HO–CβCα / deg [b]		83.9	72.4	84.2
OCβ–CαC1 / deg		-61.6	-65.1	-61.9
HBond acceptor				
HS–CβCα / deg		-65.0	-54.9	75.3
SCβ–CαC1 / deg		63.9	66.6	-67.8
r(H···S) / Å		2.356	2.446	2.372
∠(O–H···S) / deg		172.0	156.0	175.7
r(S–H···centroid) / Å		2.602	2.602	2.537
Energetic parameters				
ΔE/ kJ mol^{-1} [c]		0.0	0.1	1.1
ΔG / kJ mol^{-1}		0.0	1.4	1.6
Ec / kJ mol^{-1}		−49.1	−45.2	−47.3
ΔEc / kJ mol^{-1}		0.0	3.9	1.8
N [d]	158			
σ / kHz	6.4			

[a]Rotational constants (A, B, C), Watson's S-reduction centrifugal distortion constants (D_J, D_{JK}, D_K, d_1, d_2) and electric dipole moments (μ_α, α = a, b, c). [b]Structural parameters of the dimer. [c]Relative electronic energies (ΔE) with zero-point correction, Gibbs energy (ΔG, 298K, 1 atm), complexation energies (E_c) and relative complexation energies (ΔE_c). [d]Number of transitions (N) and rms deviation (σ) of the fit. [e]Standard errors in units of the last digit.

Table S3. Continued.

	Isomer 4	Isomer 5	Isomer 6
	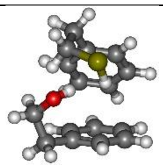 Ggπ+Ggπ–Lp–	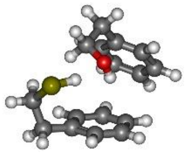 Ggπ+Ggπ–Lp+	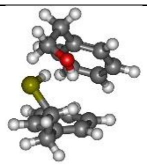 Ggπ–Ggπ–Lp+
<u>*Rotational parameters*</u>			
A / MHz[a]	551.3	614.7	673.1
B / MHz	275.2	246.3	228.3
C / MHz	234.8	213.5	196.6
D_J / kHz	0.0188	0.0114	0.0076
D_{JK} / kHz	−0.0487	−0.0126	0.0136
D_K / kHz	0.1953	0.1280	0.0506
d_1 / kHz	-0.0015	-0.0015	-0.0013
d_2 / kHz	-0.0002	-0.0001	-0.0001
$\|\mu_a\|$ / D	2.5	1.9	1.6
$\|\mu_b\|$ / D	0.7	1.0	1.7
$\|\mu_c\|$ / D	1.5	1.9	0.2
μ_{TOTAL} / D	3.0	2.9	2.3
<u>*Structural parameters*</u>			
HBond donor:			
HO–CβCα / deg [b]	75.6		
OCβ–CαC1 / deg	-63.9		
HS–CβCα / deg		70.8	-75.4
SCβ–CαC1 / deg		-66.0	63.7
HBond acceptor:			
HS–CβCα / deg	-72.8		
SCβ–CαC1 / deg	70.1		
HO–CβCα / deg		-75.6	-77.5
OCβ–CαC1 / deg		65.9	64.5
r(H···S) / Å	2.402		
∠(O–H···S) / deg	165.5		
r(H···O) / Å		2.165	2.126
∠(S–H···O) / deg		161.4	179.1
r(S–H···centroid) / Å	2.493		
r(O–H···centroid) / Å		2.401	2.414
<u>*Energetic parameters*</u>			
ΔE/ kJ mol^{-1}[c]	1.3	3.7	4.9
ΔG / kJ mol^{-1}	1.9	5.1	3.9
Ec / kJ mol^{-1}	-45.9	−41.9	−41.6
ΔEc / kJ mol^{-1}	3.2	7.2	7.5

[a]Rotational constants (*A*, *B*, *C*), Watson's S-reduction centrifugal distortion constants (D_J, D_{JK}, D_K, d_1, d_2) and electric dipole moments (μ_α, α = a, b, c). [b]Structural parameters of the dimer. [c]Relative electronic energies (Δ*E*) with zero-point correction, Gibbs energy (Δ*G*, 298K, 1 atm), complexation energies (E_c) and relative complexation energies (ΔE_c). [d]Number of transitions (*N*) and rms deviation (*σ*) of the fit. [e]Standard errors in units of the last digit.

Table S3. Continued.

	Isomer 7	Isomer 8
	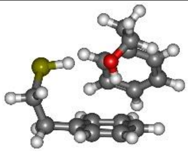 Ggπ−Ggπ+Lp−	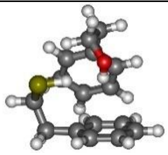 Ggπ+Ggπ−Lp−
Rotational parameters		
A / MHz[a]	491.3	458.8
B / MHz	284.8	306.3
C / MHz	255.5	267.3
D_J / kHz	0.0251	0.0208
D_{JK} / kHz	−0.0047	0.0577
D_K / kHz	0.0192	-0.0478
d_1 / kHz	-0.0018	0.0008
d_2 / kHz	0.0003	-0.0011
$\|\mu_a\|$ / D	0.6	2.1
$\|\mu_b\|$ / D	1.8	0.8
$\|\mu_c\|$ / D	0.3	1.3
μ_{TOTAL} / D	2.0	2.6
Structural parameters		
HBond donor:		
HO−CβCα / deg [b]		
OCβ−CαC1 / deg		
HS−CβCα / deg	-78.1	70.0
SCβ−CαC1 / deg	67.0	-65.9
HBond acceptor:		
HS−CβCα / deg		
SCβ−CαC1 / deg		
HO−CβCα / deg	77.2	-56.2
OCβ−CαC1 / deg	-64.4	65.6
r(H···S) / Å		
∠(O−H···S) / deg		
r(H···O) / Å	2.108	2.367
∠(S−H···O) / deg	174.8	147.1
r(S−H···centroid) / Å		
r(O−H···centroid) / Å	2.383	2.600
Energetic parameters		
ΔE/ kJ mol^{-1}[c]	4.8	6.2
ΔG / kJ mol^{-1}	4.3	7.1
Ec / kJ mol^{-1}	−42.5	−39.3
ΔEc / kJ mol^{-1}	6.6	9.8

[a]Rotational constants (*A*, *B*, *C*), Watson's S-reduction centrifugal distortion constants (D_J, D_{JK}, D_K, d_1, d_2) and electric dipole moments (μ_α, α = a, b, c). [b]Structural parameters of the dimer. [c]Relative electronic energies (Δ*E*) with zero-point correction, Gibbs energy (Δ*G*, 298K, 1 atm), complexation energies (E_c) and relative complexation energies (ΔE_c). [d]Number of transitions (*N*) and rms deviation (*σ*) of the fit. [e]Standard errors in units of the last digit.

Table S4. Conformational search for the heterodimer of 2-phenylethanethiol and 2-phenylethanol (PET-PEAL) using the B2PLYP-D3(BJ) method (tight convergence, ultrafine integration) and the def2-TZVP basis set, and comparison with the experimental rotational parameters.

	Experiment	Isomer 1	Isomer 2	Isomer 3
		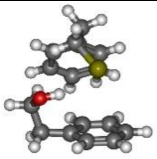 Ggπ+Ggπ–Lp+	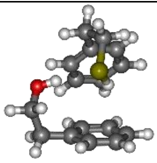 Ggπ+Ggπ–Lp–	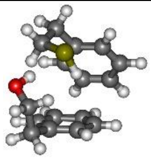 Ggπ+Ggπ+Lp+
<u>*Rotational parameters*</u>				
A / MHz [a]	456.19818(99) [e]	476.2	459.5	616.3
B / MHz	308.65432(26)	304.8	315.6	255.9
C / MHz	281.92839(29)	279.3	287.3	214.1
D_J / kHz	0.0256(10)	0.0380	0.0230	0.0119
D_{JK} / kHz	0.1077(51)	0.0603	-0.1027	0.1216
D_K / kHz		-0.0484	0.0981	-0.0050
d_1 / kHz		0.0009	-0.0004	-0.0024
d_2 / kHz		-0.0007	-0.0009	-0.0002
$\|\mu_a\|$ / D	++	0.8	2.1	1.8
$\|\mu_b\|$ / D	+	0.3	1.3	1.0
$\|\mu_c\|$ / D		1.2	0.2	0.1
μ_{TOTAL} / D		1.4	2.5	2.1
<u>*Structural parameters*</u>				
HBond donor				
HO–CβCα / deg [b]		83.6	71.7	84.2
OCβ–CαC1 / deg		-61.4	-65.0	-61.8
HBond acceptor				
HS–CβCα / deg		-65.4	-55.7	75.3
SCβ–CαC1 / deg		63.8	66.5	-67.7
r(H···S) / Å		2.376	2.466	2.392
∠(O–H···S) / deg		171.3	155.8	175.1
r(S–H···centroid) / Å		2.607	2.596	2.531
<u>*Energetic parameters*</u>				
ΔE/ kJ mol^{-1} [c]		0.0	0.1	1.2
ΔG / kJ mol^{-1}		0.0	1.4	1.6
*E*c / kJ mol^{-1}		-42.7	-38.8	-41.3
ΔEc / kJ mol^{-1}		0.0	3.9	1.4
N [d]	158			
σ / kHz	6.4			

[a]Rotational constants (*A*, *B*, *C*), Watson's S-reduction centrifugal distortion constants (D_J, D_{JK}, D_K, d_1, d_2) and electric dipole moments (μ_α, α = a, b, c). [b]Structural parameters of the dimer. [c]Relative electronic energies (Δ*E*) with zero-point correction, Gibbs energy (Δ*G*, 298K, 1 atm), complexation energies (E_c) and relative complexation energies (ΔE_c). [d]Number of transitions (*N*) and rms deviation (σ) of the fit. [e]Standard errors in units of the last digit.

Table S4. Continued.

	Isomer 4	Isomer 5	Isomer 6
	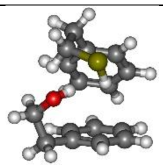	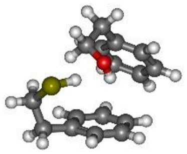	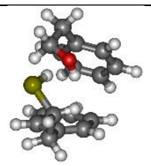
	Ggπ+Ggπ−Lp−	Ggπ+Ggπ−Lp+	Ggπ−Ggπ−Lp+
Rotational parameters			
A / MHz[a]	549.5	613.1	672.8
B / MHz	274.9	246.3	228.1
C / MHz	234.5	213.4	196.5
D_J / kHz	0.0217	0.0120	0.0080
D_{JK} / kHz	0.2443	0.1340	0.0539
D_K / kHz	-0.063303	-0.0154	0.0132
d_1 / kHz	-0.0016	-0.0015	-0.0013
d_2 / kHz	-0.0003	-0.0001	-0.0001
$\lvert\mu_a\rvert$ / D	2.5	1.9	1.6
$\lvert\mu_b\rvert$ / D	0.7	1.0	1.6
$\lvert\mu_c\rvert$ / D	1.5	1.9	0.2
μ_{TOTAL} / D	3.0	2.9	2.3
Structural parameters			
HBond donor:			
HO–CβCα / deg [b]	75.9		
OCβ–CαC1 / deg	-63.8		
HS–CβCα / deg		70.7	-75.3
SCβ–CαC1 / deg		-65.9	63.9
HBond acceptor:			
HS–CβCα / deg	-72.5		
SCβ–CαC1 / deg	69.7		
HO–CβCα / deg		-75.2	-77.2
OCβ–CαC1 / deg		65.6	64.3
r(H···S) / Å	2.421		
∠(O–H···S) / deg	165.4		
r(H···O) / Å		2.178	2.138
∠(S–H···O) / deg		161.5	178.9
r(S–H···centroid) / Å	2.488		
r(O–H···centroid) / Å		2.405	2.411
Energetic parameters			
ΔE/ kJ mol^{-1}[c]	1.4	2.7	3.7
ΔG / kJ mol^{-1}	1.5	4.1	2.9
Ec / kJ mol^{-1}	-39.7	-36.9	-37.0
ΔEc / kJ mol^{-1}	3.0	5.8	5.7

[a]Rotational constants (A, B, C), Watson's S-reduction centrifugal distortion constants (D_J, D_{JK}, D_K, d_1, d_2) and electric dipole moments (μ_α, α = a, b, c). [b]Structural parameters of the dimer. [c]Relative electronic energies (ΔE) with zero-point correction, Gibbs energy (ΔG, 298K, 1 atm), complexation energies (E_c) and relative complexation energies (ΔE_c). [d]Number of transitions (N) and rms deviation (σ) of the fit. [e]Standard errors in units of the last digit.

Table S4. Continued.

	Isomer 7	Isomer 8
	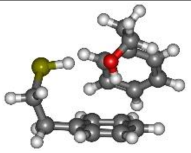 Ggπ–Ggπ+Lp–	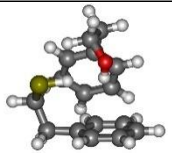 Ggπ+Ggπ–Lp–
Rotational parameters		
A / MHz[a]	493.9	459.2
B / MHz	282.1	305.1
C / MHz	254.5	266.5
D_J / kHz	0.0290	0.0211
D_{JK} / kHz	0.0235	-0.0464
D_K / kHz	-0.0077	0.0555
d_1 / kHz	-0.0024	0.0007
d_2 / kHz	0.0007	-0.0011
$\|\mu_a\|$ / D	0.6	2.1
$\|\mu_b\|$ / D	1.9	0.8
$\|\mu_c\|$ / D	0.3	1.3
μ_{TOTAL} / D	2.0	2.6
Structural parameters		
HBond donor:		
HO–CβCα / deg [b]		
OCβ–CαC1 / deg		
HS–CβCα / deg	-78.7	69.9
SCβ–CαC1 / deg	67.2	-65.9
HBond acceptor:		
HS–CβCα / deg		
SCβ–CαC1 / deg		
HO–CβCα / deg	79.0	-56.3
OCβ–CαC1 / deg	-63.8	65.6
r(H···S) / Å		
∠(O–H···S) / deg		
r(H···O) / Å	2.128	2.368
∠(S–H···O) / deg	173.8	147.0
r(S–H···centroid) / Å		
r(O–H···centroid) / Å	2.352	2.623
Energetic parameters		
ΔE/ kJ mol^{-1}[c]	3.9	5.6
ΔG / kJ mol^{-1}	2.7	6.6
E_c / kJ mol^{-1}	-37.3	-33.9
ΔE_c / kJ mol^{-1}	5.4	8.9

[a]Rotational constants (A, B, C), Watson's S-reduction centrifugal distortion constants (D_J, D_{JK}, D_K, d_1, d_2) and electric dipole moments (μ_α, α = a, b, c). [b]Structural parameters of the dimer. [c]Relative electronic energies (ΔE) with zero-point correction, Gibbs energy (ΔG, 298K, 1 atm), complexation energies (E_c) and relative complexation energies (ΔE_c). [d]Number of transitions (N) and rms deviation (σ) of the fit. [e]Standard errors in units of the last digit.

Table S5. Atomic coordinates (principal inertial axes system) for isomer 1 of the homodimer of 2-phenylethanethiol (PET-PET) according to B2PLYP-D3(BJ)/def2-TZVP calculations.

Atom	*a* / Å	*b* / Å	*c* / Å
C	-2.7577	-0.8628	-0.5986
C	-2.5626	0.4879	-0.3098
C	-2.4652	0.8738	1.0299
C	-2.5679	-0.0619	2.0527
C	-2.7729	-1.4057	1.7511
C	-2.8660	-1.8030	0.4219
C	-2.4474	1.5118	-1.4071
C	-1.0149	2.0004	-1.6333
S	0.1207	0.7344	-2.2850
H	-2.8298	-1.1773	-1.6321
H	-2.3214	1.9203	1.2728
H	-2.5000	0.2582	3.0844
H	-2.8638	-2.1345	2.5457
H	-3.0281	-2.8450	0.1781
H	-3.0601	2.3817	-1.1516
H	-2.8377	1.1012	-2.3391
H	-1.0065	2.7888	-2.3868
H	-0.5955	2.4199	-0.7189
H	0.0161	-0.1286	-1.2638
C	0.7965	-1.7122	0.7046
C	1.9316	-0.9197	0.8935
C	2.9327	-0.9463	-0.0779
C	2.8032	-1.7431	-1.2116
C	1.6725	-2.5342	-1.3856
C	0.6688	-2.5179	-0.4213
C	2.0383	-0.0287	2.1007
C	1.2837	1.2940	1.9343
S	2.0123	2.4435	0.7233
H	0.0042	-1.6971	1.4428
H	3.8152	-0.3334	0.0530
H	3.5866	-1.7466	-1.9579
H	1.5732	-3.1563	-2.2650
H	-0.2175	-3.1253	-0.5473
H	3.0852	0.1834	2.3219
H	1.6173	-0.5461	2.9677
H	0.2427	1.1058	1.6741
H	1.2947	1.8487	2.8737
H	1.7893	1.6993	-0.3739

Table S6. Atomic coordinates (principal inertial axes system) for isomer 1 of the heterodimer of 2-phenylethanethiol and 2-phenylethanol (PET-PEAL) according to B2PLYP-D3(BJ)/def2-TZVP calculations.

Atom	*a* / Å	*b* / Å	*c* / Å
C	-1.8854	0.0842	-1.4354
C	-2.3514	0.6267	-0.2386
C	-3.0495	-0.2031	0.6434
C	-3.2808	-1.5381	0.3354
C	-2.8229	-2.0658	-0.8692
C	-2.1260	-1.2499	-1.7523
C	-2.1232	2.0810	0.0880
C	-1.3772	2.3198	1.3999
S	0.3288	1.6775	1.4341
H	-1.3192	0.7055	-2.1175
H	-3.4269	0.1997	1.5759
H	-3.8265	-2.1641	1.0294
H	-3.0100	-3.1028	-1.1154
H	-1.7643	-1.6509	-2.6904
H	-3.0907	2.5897	0.1604
H	-1.5760	2.5546	-0.7282
H	-1.9203	1.9034	2.2470
H	-1.2714	3.3902	1.5785
H	0.0262	0.3736	1.3425
C	0.6718	-2.0999	0.5928
C	1.3183	-1.9687	1.8178
C	2.4953	-1.2287	1.8973
C	3.0190	-0.6272	0.7584
C	2.3841	-0.7574	-0.4799
C	1.2068	-1.5018	-0.5445
C	2.9241	-0.0658	-1.7015
C	2.5510	1.4193	-1.7546
O	1.1500	1.6270	-1.8029
H	-0.2520	-2.6581	0.5188
H	0.9076	-2.4343	2.7039
H	3.0035	-1.1189	2.8462
H	3.9343	-0.0513	0.8301
H	0.6965	-1.6025	-1.4923
H	2.5381	-0.5498	-2.6004
H	4.0149	-0.1449	-1.7274
H	2.9870	1.9401	-0.8952
H	2.9682	1.8636	-2.6597
H	0.8220	1.5932	-0.8888

Table S7. Atomic coordinates (principal inertial axes system) for isomer 2 of the of the heterodimer of 2-phenylethanethiol and 2-phenylethanol (PET-PEAL) according to B2PLYP-D3(BJ)/def2-TZVP calculations.

Atom	*a* / Å	*b* / Å	*c* / Å
C	-2.5174	-0.6955	-1.0220
C	-2.4741	0.4659	-0.2512
C	-2.5123	0.3424	1.1405
C	-2.5953	-0.9060	1.7449
C	-2.6449	-2.0578	0.9636
C	-2.6061	-1.9481	-0.4215
C	-2.3720	1.8243	-0.8925
C	-0.9917	2.4638	-0.7327
S	0.3457	1.5976	-1.6192
H	-2.4848	-0.6160	-2.1014
H	-2.4897	1.2346	1.7553
H	-2.6317	-0.9807	2.8239
H	-2.7183	-3.0304	1.4318
H	-2.6491	-2.8373	-1.0371
H	-3.1048	2.4954	-0.4345
H	-2.6148	1.7530	-1.9534
H	-0.9927	3.4682	-1.1571
H	-0.7227	2.5561	0.3189
H	0.1619	0.3885	-1.0660
C	0.9921	-2.1524	-0.8267
C	1.9878	-1.8584	-1.7541
C	3.0327	-1.0142	-1.3933
C	3.0822	-0.4705	-0.1133
C	2.0869	-0.7530	0.8233
C	1.0402	-1.5996	0.4484
C	2.0927	-0.0985	2.1762
C	1.2661	1.1911	2.1939
O	1.8001	2.2070	1.3647
H	0.1734	-2.8059	-1.0958
H	1.9494	-2.2837	-2.7481
H	3.8101	-0.7782	-2.1081
H	3.8952	0.1896	0.1615
H	0.2512	-1.8220	1.1563
H	1.6772	-0.7803	2.9231
H	3.1140	0.1419	2.4767
H	0.2316	0.9570	1.9159
H	1.2555	1.6018	3.2045
H	1.6586	1.9602	0.4368

Table S8. List of observed rotational transitions of the homodimer of 2-phenylethanethiol (PET-PET) and differences with the predicted transitions with the parameters of Table 1.

	J'	K_{-1}'	K_{+1}'	*J"*	K_{-1}''	K_{+1}''	Freq. / MHz	o.-c. / MHz
1	4	1	4	3	1	3	2128.2668	0.0025
2	4	0	4	3	0	3	2148.9610	0.0092
3	4	1	3	3	1	2	2198.1424	-0.0107
4	4	2	2	3	1	2	2501.5400	-0.0027
5	5	1	5	4	1	4	2657.5036	0.0024
6	5	0	5	4	0	4	2675.5574	0.0052
7	5	2	4	4	2	3	2704.5720	-0.0125
8	5	1	5	4	0	4	2712.1980	0.0111
9	5	3	3	4	3	2	2714.2992	0.0165
10	5	3	2	4	3	1	2717.0477	0.0070
11	5	2	3	4	2	2	2737.8647	-0.0093
12	5	1	4	4	1	3	2743.1051	-0.0025
13	6	1	6	5	1	5	3185.4209	-0.0053
14	6	0	6	5	0	5	3199.1103	-0.0034
15	6	2	5	5	2	4	3242.0071	0.0096
16	6	5	2	5	5	1	3255.5406	0.0050
17	6	5	1	5	5	0	3255.5406	0.0010
18	6	4	3	5	4	2	3257.1776	-0.0080
19	6	4	2	5	4	1	3257.4877	0.0277
20	6	3	4	5	3	3	3257.9914	-0.0196
21	6	3	3	5	3	2	3265.1361	0.0043
22	6	1	5	5	1	4	3284.3309	-0.0107
23	6	2	4	5	2	3	3293.2799	-0.0056
24	5	3	2	4	2	2	3296.2978	-0.0035
25	5	4	1	4	3	1	3548.9921	-0.0223
26	5	4	2	4	3	2	3549.8796	-0.0019
27	6	2	4	5	1	4	3591.4350	-0.0064
28	7	1	7	6	1	6	3712.1941	-0.0082
29	7	0	7	6	0	6	3721.5064	0.0015
30	7	1	7	6	0	6	3735.1376	-0.0120
31	6	2	5	5	1	5	3740.5499	0.0293
32	7	2	6	6	2	5	3777.6208	-0.0051
33	5	5	1	4	4	0	3788.9913	0.0055
34	5	5	0	4	4	0	3788.9913	0.0051
35	5	5	1	4	4	1	3788.9913	-0.0021
36	5	5	0	4	4	1	3788.9913	-0.0025
37	7	6	2	6	6	1	3797.8735	0.0005
38	7	6	1	6	6	0	3797.8735	0.0002
39	7	5	3	6	5	2	3799.3783	0.0299
40	7	5	2	6	5	1	3799.3783	0.0081
41	7	3	5	6	3	4	3801.4189	0.0031
42	7	4	4	6	4	3	3801.7561	-0.0131
43	7	4	3	6	4	2	3802.6742	0.0047
44	6	3	4	5	2	3	3812.7030	-0.0410
45	7	3	4	6	3	3	3816.5523	-0.0154
46	7	1	6	6	1	5	3820.7334	-0.0118
47	6	3	3	5	2	3	3823.5786	0.0195
48	7	2	5	6	2	4	3847.6624	-0.0029
49	6	3	4	5	2	4	3873.7796	0.0081
50	7	2	6	6	1	5	3972.4270	0.0168
51	6	4	3	5	3	2	4089.0849	-0.0053
52	6	4	2	5	3	2	4089.4340	0.0002

53	6	4	3	5	3	3	4092.7829	-0.0016
54	6	4	2	5	3	3	4093.1223	-0.0057
55	7	2	5	6	1	5	4154.7569	-0.0080
56	8	1	7	7	2	6	4199.9892	0.0070
57	7	1	6	6	0	6	4204.0218	0.0192
58	8	0	8	7	1	7	4230.2581	-0.0006
59	8	1	8	7	1	7	4238.0640	0.0004
60	8	0	8	7	0	7	4243.8991	-0.0044
61	8	1	8	7	0	7	4251.7125	0.0041
62	8	2	7	7	2	6	4311.3349	-0.0022
63	7	3	5	6	2	4	4320.8798	0.0055
64	6	5	1	5	4	2	4331.3424	0.0142
65	7	2	6	6	1	6	4332.7275	0.0073
66	8	7	1	7	7	0	4340.2151	-0.0035
67	8	7	2	7	7	1	4340.2151	-0.0035
68	8	6	3	7	6	2	4341.5393	0.0051
69	8	6	2	7	6	1	4341.5393	0.0036
70	8	5	4	7	5	3	4343.7137	-0.0026
71	8	5	3	7	5	2	4343.8133	0.0105
72	8	3	6	7	3	5	4344.0322	0.0076
73	8	4	5	7	4	4	4346.8875	-0.0023
74	8	4	4	7	4	3	4349.3059	0.0004
75	8	1	7	7	1	6	4351.6577	0.0107
76	8	3	5	7	3	4	4371.7400	-0.0092
77	8	2	6	7	2	5	4399.2265	0.0018
78	7	3	5	6	2	5	4433.1978	0.0080
79	7	3	4	6	2	5	4459.1415	-0.0152
80	6	6	1	5	5	0	4570.7165	-0.0006
81	6	6	0	5	5	0	4570.7165	-0.0006
82	6	6	1	5	5	1	4570.7165	-0.0010
83	6	6	0	5	5	1	4570.7165	-0.0010
84	7	4	3	6	3	3	4626.9631	-0.0082
85	7	4	4	6	3	4	4636.5382	-0.0046
86	7	4	3	6	3	4	4637.7972	0.0108
87	8	2	6	7	1	6	4733.2351	-0.0093
88	9	0	9	8	1	8	4758.9184	0.0006
89	9	1	9	8	1	8	4763.2531	-0.0016
90	9	1	8	8	2	7	4766.0801	0.0153
91	9	0	9	8	0	8	4766.7184	-0.0041
92	9	1	9	8	0	8	4771.0526	-0.0068
93	8	3	6	7	2	5	4817.2401	0.0067
94	9	2	8	8	2	7	4843.1212	0.0085
95	8	3	5	7	2	5	4870.9305	0.0053
96	7	5	3	6	4	2	4873.1783	0.0353
97	7	5	2	6	4	2	4873.1783	0.0091
98	7	5	3	6	4	3	4873.4625	-0.0239
99	7	5	2	6	4	3	4873.4625	-0.0501
100	9	1	8	8	1	7	4877.4288	0.0090
101	9	8	1	8	8	0	4882.5637	0.0009
102	9	8	2	8	8	1	4882.5637	0.0009
103	9	7	3	8	7	2	4883.7687	-0.0027
104	9	7	2	8	7	1	4883.7687	-0.0028
105	9	3	7	8	3	6	4885.3774	0.0080
106	9	6	4	8	6	3	4885.6428	0.0008
107	9	6	3	8	6	2	4885.6428	-0.0061
108	9	5	5	8	5	4	4888.6989	0.0027
109	9	5	4	8	5	3	4888.9645	-0.0083
110	9	4	6	8	4	5	4892.3621	-0.0042
111	9	4	5	8	4	4	4897.9598	0.0068
112	9	3	6	8	3	5	4929.9990	-0.0025

113	8	2	7	7	1	7	4931.8713	0.0163
114	9	2	7	8	2	6	4946.7611	-0.0015
115	9	2	8	8	1	7	4954.4851	0.0173
116	8	3	6	7	2	6	4999.5841	-0.0041
117	10	2	8	9	3	6	5034.3866	0.0035
118	7	6	2	6	5	1	5113.0453	-0.0053
119	7	6	1	6	5	1	5113.0453	-0.0055
120	7	6	2	6	5	2	5113.0453	-0.0097
121	7	6	1	6	5	2	5113.0453	-0.0099
122	8	4	5	7	3	4	5156.0528	0.0029
123	8	4	4	7	3	4	5159.7356	0.0264
124	8	4	5	7	3	5	5182.0191	0.0021
125	8	4	4	7	3	5	5185.6605	-0.0156
126	13	7	7	12	8	4	5276.0583	-0.0122
127	13	7	7	12	8	5	5276.0583	-0.0124
128	13	7	6	12	8	4	5276.0583	-0.0461
129	13	7	6	12	8	5	5276.0583	-0.0463
130	10	0	10	9	1	9	5285.6346	0.0031
131	10	1	10	9	1	9	5287.9835	-0.0049
132	10	0	10	9	0	9	5289.9661	-0.0021
133	10	1	10	9	0	9	5292.3294	0.0040
134	10	1	9	9	2	8	5322.5624	-0.0125
135	9	2	7	8	1	7	5328.3365	-0.0235
136	7	7	0	6	6	0	5352.4382	0.0003
137	7	7	0	6	6	1	5352.4382	0.0003
138	7	7	1	6	6	0	5352.4382	0.0003
139	7	7	1	6	6	1	5352.4382	0.0003
140	10	1	9	9	1	8	5399.6291	0.0062
141	9	3	6	8	2	6	5401.6980	-0.0041
142	8	5	4	7	4	3	5414.1938	0.0039
143	8	5	3	7	4	3	5414.3018	-0.0007
144	8	5	4	7	4	4	5415.4307	-0.0028
145	8	5	3	7	4	4	5415.5691	0.0229
146	10	9	2	9	9	1	5424.8903	-0.0085
147	10	3	8	9	3	7	5425.0567	0.0122
148	10	8	2	9	8	1	5426.0402	0.0053
149	10	8	3	9	8	2	5426.0402	0.0053
150	10	7	4	9	7	3	5427.6951	0.0057
151	10	7	3	9	7	2	5427.6951	0.0052
152	10	6	5	9	6	4	5430.2673	0.0133
153	10	6	4	9	6	3	5430.2673	-0.0126
154	10	5	6	9	5	5	5434.3189	0.0057
155	10	5	5	9	5	4	5435.0702	-0.0020
156	10	4	7	9	4	6	5437.8987	0.0052
157	10	4	6	9	4	5	5449.3470	-0.0044
158	10	2	9	9	1	8	5450.1075	0.0023
159	12	4	9	11	5	6	5465.5788	-0.0422
160	9	1	8	8	0	8	5467.6776	0.0166
161	10	2	8	9	2	7	5489.3223	-0.0003
162	10	3	7	9	3	6	5489.4033	-0.0029
163	9	3	7	8	2	7	5573.6162	-0.0042
164	8	6	3	7	5	2	5655.2283	0.0137
165	8	6	2	7	5	2	5655.2283	0.0119
166	8	6	3	7	5	3	5655.2283	-0.0124
167	8	6	2	7	5	3	5655.2283	-0.0142
168	9	4	6	8	3	5	5676.6543	-0.0126
169	9	4	5	8	3	5	5685.9088	-0.0041
170	9	4	6	8	3	6	5730.3520	-0.0067
171	9	4	5	8	3	6	5739.5974	-0.0072
172	11	0	11	10	1	10	5811.1626	-0.0059

173	11	1	11	10	1	10	5812.4155	-0.0119
174	11	0	11	10	0	10	5813.5138	-0.0118
175	11	1	11	10	0	10	5814.7889	0.0043
176	11	1	10	10	2	9	5869.8133	0.0168
177	15	9	7	14	10	5	5878.6707	0.0100
178	15	9	6	14	10	4	5878.6707	0.0098
179	8	7	1	7	6	1	5894.7768	-0.0063
180	8	7	2	7	6	1	5894.7768	-0.0063
181	8	7	1	7	6	2	5894.7768	-0.0066
182	8	7	2	7	6	2	5894.7768	-0.0066
183	11	2	10	10	2	9	5901.3894	0.0055
184	11	1	10	10	1	9	5920.2864	0.0076
185	10	2	8	9	1	8	5940.2361	-0.0267
186	10	3	7	9	2	7	5944.3321	-0.0136
187	9	5	5	8	4	4	5953.5762	-0.0043
188	9	5	4	8	4	4	5953.9760	0.0060
189	9	5	5	8	4	5	5957.2318	-0.0079
190	9	5	4	8	4	5	5957.6196	-0.0096
191	11	3	9	10	3	8	5962.7554	0.0101
192	11	9	2	10	9	1	5968.3186	0.0110
193	11	9	3	10	9	2	5968.3186	0.0110
194	11	8	3	10	8	2	5969.8142	0.0000
195	11	8	4	10	8	3	5969.8142	0.0000
196	11	7	5	10	7	4	5972.0158	0.0000
197	11	7	4	10	7	3	5972.0158	-0.0021
198	11	6	6	10	6	5	5975.4067	-0.0187
199	11	6	5	10	6	4	5975.5083	0.0009
200	11	5	7	10	5	6	5980.5416	0.0019
201	11	5	6	10	5	5	5982.3847	0.0007
202	11	4	8	10	4	7	5983.0649	0.0056
203	11	4	7	10	4	6	6004.2050	-0.0155
204	11	2	9	10	2	8	6026.0805	0.0035
205	11	3	8	10	3	7	6047.4849	-0.0019
206	8	8	0	7	7	0	6134.1510	-0.0012
207	8	8	0	7	7	1	6134.1510	-0.0012
208	8	8	1	7	7	0	6134.1510	-0.0012
209	8	8	1	7	7	1	6134.1510	-0.0012
210	10	4	6	9	3	6	6205.2698	0.0070
211	10	4	7	9	3	7	6282.8979	0.0149
212	10	4	6	9	3	7	6303.5987	0.0119
213	12	1	12	11	1	11	6336.6825	-0.0018
214	12	2	11	11	2	10	6428.3798	0.0037
215	9	7	3	8	6	2	6437.0154	-0.0034
216	9	7	2	8	6	2	6437.0154	-0.0036
217	9	7	3	8	6	3	6437.0154	-0.0052
218	9	7	2	8	6	3	6437.0154	-0.0053
219	12	1	11	11	1	10	6440.9268	0.0105
220	10	5	5	9	4	5	6491.0765	-0.0126
221	12	3	10	11	3	9	6498.3171	0.0242
222	10	5	6	9	4	6	6499.1831	-0.0034
223	12	10	3	11	10	2	6510.5899	0.0111
224	12	9	3	11	9	2	6511.9804	0.0000
225	12	9	4	11	9	3	6511.9804	0.0000
226	12	8	5	11	8	4	6513.9378	0.0044
227	12	8	4	11	8	3	6513.9378	0.0042
228	12	7	6	11	7	5	6516.8083	0.0120
229	12	7	5	11	7	4	6516.8083	0.0045
230	12	6	7	11	6	6	6521.2099	0.0055
231	12	6	6	11	6	5	6521.4310	-0.0011
232	12	5	8	11	5	7	6527.2722	0.0015

233	12	4	9	11	4	8	6527.3884	0.0010
234	12	5	7	11	5	6	6531.3230	-0.0017
235	12	2	10	11	2	9	6556.6148	-0.0008
236	12	4	8	11	4	7	6562.8376	-0.0005
237	12	3	9	11	3	8	6602.1343	0.0099
238	9	8	1	8	7	1	6676.4902	-0.0060
239	9	8	1	8	7	2	6676.4902	-0.0060
240	9	8	2	8	7	1	6676.4902	-0.0060
241	9	8	2	8	7	2	6676.4902	-0.0060
242	11	4	8	10	3	8	6840.9012	0.0034
243	13	1	13	12	0	12	6861.5079	0.0137
244	9	9	0	8	8	0	6915.8541	-0.0064
245	9	9	0	8	8	1	6915.8541	-0.0064
246	9	9	1	8	8	0	6915.8541	-0.0064
247	9	9	1	8	8	1	6915.8541	-0.0064
248	13	2	12	12	2	11	6954.3431	0.0035
249	13	1	12	12	1	11	6962.2478	0.0156
250	10	7	4	9	6	3	6979.0691	0.0098
251	10	7	3	9	6	3	6979.0691	0.0092
252	10	7	4	9	6	4	6979.0691	0.0011
253	10	7	3	9	6	4	6979.0691	0.0005
254	14	2	12	13	3	10	7009.8219	-0.0399
255	11	5	7	10	4	6	7021.0960	-0.0328
256	11	5	6	10	4	6	7024.1475	0.0257
257	11	5	7	10	4	7	7041.8285	-0.0042
258	13	12	1	12	12	0	7051.7990	-0.0203
259	13	12	2	12	12	1	7051.7990	-0.0203
260	13	8	6	12	8	5	7058.4413	0.0145
261	13	8	5	12	8	4	7058.4413	0.0139
262	13	7	7	12	7	6	7062.0858	0.0091
263	13	7	6	12	7	5	7062.0858	-0.0144
264	13	6	8	12	6	7	7067.6399	0.0185
265	13	6	7	12	6	6	7068.1904	-0.0026
266	13	4	10	12	4	9	7070.4079	0.0132
267	13	5	9	12	5	8	7074.3134	0.0117
268	13	2	11	12	2	10	7081.4665	0.0137
269	13	5	8	12	5	7	7082.4633	-0.0106
270	13	4	9	12	4	8	7124.4664	-0.0205
271	13	3	10	12	3	9	7151.8748	0.0122
272	12	2	10	11	1	10	7203.0467	-0.0073
273	10	8	2	9	7	2	7218.7533	-0.0063
274	10	8	3	9	7	2	7218.7533	-0.0063
275	10	8	2	9	7	3	7218.7533	-0.0064
276	10	8	3	9	7	3	7218.7533	-0.0064
277	11	6	5	10	5	5	7278.8208	0.0161
278	11	6	6	10	5	6	7279.8315	-0.0050
279	10	9	1	9	8	1	7458.2026	0.0060
280	10	9	1	9	8	2	7458.2026	0.0060
281	10	9	2	9	8	1	7458.2026	0.0060
282	10	9	2	9	8	2	7458.2026	0.0060
283	12	4	8	11	3	9	7482.8512	-0.0045
284	11	7	4	10	6	5	7520.8187	-0.0139
285	11	7	5	10	6	5	7520.8193	-0.0106
286	10	10	0	9	9	0	7697.5730	0.0106
287	10	10	0	9	9	1	7697.5730	0.0106
288	10	10	1	9	9	0	7697.5730	0.0106
289	10	10	1	9	9	1	7697.5730	0.0106

Table S9. List of observed rotational transitions of the heterodimer of 2-phenylethanethiol and 2-phenylethanol (PET-PEAL) and differences with the predicted transitions with the parameters of Table 1.

	J'	K_{-1}'	K_{+1}'	*J''*	K_{-1}''	K_{+1}''	Freq. / MHz	o.-c. / MHz
1	4	1	4	3	1	3	2302.9447	−0.0089
2	4	0	4	3	0	3	2331.8648	−0.0052
3	4	2	3	3	2	2	2359.7479	0.0130
4	4	2	2	3	2	1	2390.1701	−0.0022
5	4	1	3	3	1	2	2408.3137	−0.0057
6	5	1	5	4	1	4	2874.0287	−0.0052
7	5	0	5	4	0	4	2897.9778	0.0063
8	5	2	4	4	2	3	2945.5423	−0.0032
9	5	3	3	4	3	2	2961.8144	−0.0012
10	5	3	2	4	3	1	2967.0715	−0.0092
11	5	2	3	4	2	2	3000.3428	0.0011
12	5	1	4	4	1	3	3002.3932	−0.0042
13	6	1	6	5	1	5	3443.0623	−0.0035
14	6	0	6	5	0	5	3460.1569	0.0017
15	6	2	5	5	2	4	3528.7261	0.0041
16	6	4	3	5	4	2	3554.5468	0.0150
17	6	4	2	5	4	1	3555.1429	0.0185
18	6	3	4	5	3	3	3555.3575	0.0019
19	6	3	3	5	3	2	3568.8162	−0.0044
20	6	1	5	5	1	4	3589.9520	0.0053
21	6	2	4	5	2	3	3611.4454	−0.0029
22	7	1	7	6	1	6	4010.3912	0.0088
23	7	0	7	6	0	6	4021.3108	0.0061
24	7	2	6	6	2	5	4108.9296	−0.0027
25	7	6	2	6	6	1	4143.2977	−0.0321
26	7	6	1	6	6	0	4143.2977	−0.0328
27	7	3	5	6	3	4	4148.1137	0.0046
28	7	4	4	6	4	3	4149.7946	0.0095
29	7	4	3	6	4	2	4151.7138	−0.0075
30	7	1	6	6	1	5	4169.2257	−0.0003
31	7	3	4	6	3	3	4176.2948	0.0012
32	7	2	5	6	2	4	4219.7347	0.0000
33	8	1	8	7	1	7	4576.3850	−0.0179
34	8	0	8	7	0	7	4582.8700	0.0048
35	8	2	7	7	2	6	4686.0218	−0.0020
36	8	3	6	7	3	5	4739.2607	0.0010
37	8	1	7	7	1	6	4739.7017	0.0016
38	8	4	5	7	4	4	4745.8053	−0.0039
39	8	4	4	7	4	3	4750.9789	0.0110
40	8	3	5	7	3	4	4789.5551	−0.0045
41	8	2	6	7	2	5	4822.5999	−0.0053
42	9	1	9	8	1	8	5141.5324	0.0037
43	9	0	9	8	0	8	5145.1564	0.0017
44	9	2	8	8	2	7	5260.0622	0.0000
45	9	1	8	8	1	7	5302.9895	0.0122
46	9	3	7	8	3	6	5328.0473	0.0003
47	9	7	3	8	7	2	5328.5360	0.0092
48	9	7	2	8	7	1	5328.5360	0.0089
49	9	6	4	8	6	3	5331.7070	0.0036
50	9	6	3	8	6	2	5331.7070	−0.0156
51	9	5	5	8	5	4	5336.8449	0.0131

52	9	4	6	8	4	5	5342.1974	−0.0015
53	9	4	5	8	4	4	5353.9872	−0.0052
54	9	3	6	8	3	5	5406.4512	−0.0094
55	9	2	7	8	2	6	5418.2649	0.0002
56	10	1	10	9	1	9	5706.0719	−0.0091
57	10	0	10	9	0	9	5708.0388	−0.0023
58	10	2	9	9	2	8	5831.3370	0.0080
59	10	1	9	9	1	8	5862.3195	0.0035
60	10	3	8	9	3	7	5913.8656	0.0041
61	10	8	3	9	8	2	5919.9810	0.0065
62	10	8	2	9	8	1	5919.9810	0.0065
63	10	7	4	9	7	3	5922.7824	0.0032
64	10	7	3	9	7	2	5922.7824	0.0017
65	10	6	5	9	6	4	5927.1812	0.0448
66	10	6	4	9	6	3	5927.1932	−0.0143
67	10	5	6	9	5	5	5933.8689	0.0103
68	10	5	5	9	5	4	5935.7285	0.0411
69	10	4	7	9	4	6	5938.3273	−0.0058
70	10	4	6	9	4	5	5962.0976	0.0052
71	10	2	8	9	2	7	6005.1799	−0.0023
72	10	3	7	9	3	6	6023.0494	−0.0061
73	11	1	11	10	1	10	6270.2894	0.0013
74	11	0	11	10	0	10	6271.3146	−0.0044
75	11	2	10	10	2	9	6400.2752	0.0069
76	11	1	10	10	1	9	6420.8269	0.0178
77	11	3	9	10	3	8	6496.3102	0.0059
78	11	9	3	10	9	2	6511.4498	0.0005
79	11	9	2	10	9	1	6511.4498	0.0005
80	11	8	4	10	8	3	6513.9651	−0.0310
81	11	8	3	10	8	2	6513.9651	−0.0311
82	11	7	5	10	7	4	6517.7441	0.0085
83	11	7	4	10	7	3	6517.7441	0.0020
84	11	6	5	10	6	4	6523.7403	−0.0045
85	11	5	7	10	5	6	6531.7869	0.0001
86	11	4	8	10	4	7	6533.4320	−0.0032
87	11	5	6	10	5	5	6536.1857	−0.0051
88	11	4	7	10	4	6	6576.1566	−0.0058
89	11	2	9	10	2	8	6582.2721	0.0002
90	11	3	8	10	3	7	6635.3559	−0.0019
91	12	1	12	11	1	11	6834.2924	−0.0040
92	12	0	12	11	0	11	6834.8289	0.0014
93	12	2	11	11	2	10	6967.4015	0.0024
94	12	1	11	11	1	10	6980.1569	0.0082
95	12	3	10	11	3	2	7075.2263	0.0068
96	12	9	4	11	9	3	7105.3028	0.0066
97	12	9	4	11	9	2	7105.3028	0.0066
98	12	9	3	11	9	3	7105.3028	0.0066
99	12	9	3	11	9	4	7105.3028	0.0066
100	12	8	5	11	8	3	7108.5930	−0.0114
101	12	8	4	11	8	5	7108.5930	−0.0119
102	12	7	6	11	7	4	7113.4806	0.0063
103	12	7	5	11	7	6	7113.4806	−0.0169
104	12	6	7	11	6	5	7120.9192	−0.0047
105	12	6	6	11	6	8	7121.5391	−0.0035
106	12	4	9	11	4	6	7126.6770	0.0032
107	12	5	7	11	5	9	7139.8854	−0.0092
108	12	2	10	11	2	7	7149.7679	0.0115
109	12	4	8	11	4	8	7195.6754	−0.0102
110	12	3	9	11	3	2	7240.4861	−0.0079
111	13	1	13	12	1	12	7398.1881	−0.0057

112	13	0	13	12	0	12	7398.4504	−0.0124
113	13	2	12	12	2	11	7533.2284	0.0031
114	13	1	12	12	1	11	7540.7624	0.0003
115	13	3	11	12	3	10	7650.6972	−0.0033
116	13	4	10	12	4	9	7717.2756	−0.0003
117	13	6	7	12	6	6	7720.8996	−0.0055
118	13	5	9	12	5	8	7729.0297	0.0068
119	13	5	8	12	5	7	7747.9482	−0.0050
120	13	3	10	12	3	9	7836.5684	0.0018
121	14	1	14	13	1	13	7962.0242	−0.0057
122	14	0	14	13	0	13	7962.1553	−0.0091
123	3	3	1	2	2	0	2574.9087	−0.0055
124	3	3	0	2	2	1	2578.4731	−0.0123
125	5	0	5	4	1	4	2831.8882	0.0091
126	5	1	5	4	0	4	2940.0587	−0.0675
127	4	3	2	3	2	1	3158.4740	−0.0071
128	4	3	1	3	2	2	3176.5223	0.0072
129	6	0	6	5	1	5	3418.0045	0.0041
130	6	1	6	5	0	5	3485.2266	0.0059
131	5	3	3	4	2	2	3730.1183	−0.0061
132	5	3	2	4	2	3	3783.8697	0.0087
133	6	2	5	5	1	4	3828.0368	−0.0024
134	7	1	6	6	2	5	3931.1290	−0.0044
135	7	0	7	6	1	6	3996.2292	−0.0099
136	7	1	7	6	0	6	4035.4490	0.0011
137	6	3	4	5	2	3	4285.1329	−0.0054
138	6	3	3	5	2	4	4407.1436	0.0075
139	8	0	8	7	1	7	4568.7318	0.0098
140	8	1	8	7	0	7	4590.5590	0.0128
141	7	3	5	6	2	4	4821.8012	0.0020
142	8	2	7	7	1	6	4863.8225	−0.0002
143	7	3	4	6	2	5	5054.7123	0.0046
144	9	0	9	8	1	8	5137.4793	0.0055
145	9	1	9	8	0	8	5149.2214	0.0118
146	9	1	8	8	2	7	5178.8568	0.0022
147	7	2	5	6	1	6	5182.7311	0.0001
148	8	3	6	7	2	5	5341.3272	0.0030
149	9	2	8	8	1	7	5384.1829	−0.0020
150	10	2	8	9	3	7	5576.6702	−0.0107
151	10	0	10	9	1	9	5703.9618	−0.0244
152	10	1	10	9	0	9	5710.1369	0.0009
153	8	3	5	7	2	6	5735.3377	0.0027
154	10	1	9	9	2	8	5781.1260	0.0177
155	9	3	7	8	2	6	5846.7746	0.0086
156	10	2	9	9	1	8	5912.4708	−0.0659
157	11	2	9	10	3	8	6245.0886	−0.0027
158	11	0	11	10	1	10	6269.2285	0.0042
159	11	1	11	10	0	10	6272.3875	0.0046
160	11	2	10	10	1	9	6450.4862	−0.0027
161	9	3	6	8	2	7	6455.7672	−0.0045
162	11	3	9	10	2	8	6833.5176	0.0329
163	12	0	12	11	1	11	6833.7560	−0.0075
164	12	1	12	11	0	11	6835.3534	−0.0068
165	12	1	11	11	2	10	6950.4738	0.0050
166	12	2	11	11	1	10	6997.0846	0.0056
167	10	3	7	9	2	8	7218.7543	−0.0107
168	13	0	13	12	1	12	7397.9139	−0.0161
169	13	1	13	12	0	12	7398.7246	−0.0020
170	13	2	11	12	3	10	7533.2284	0.0458
171	14	1	14	13	0	13	7962.2984	0.0046
